# Characterization of MK-40 Membrane Modified by Layers of Cation Exchange and Anion Exchange Polyelectrolytes

**DOI:** 10.3390/membranes10020020

**Published:** 2020-01-27

**Authors:** Valentina Titorova, Konstantin Sabbatovskiy, Veronika Sarapulova, Evgeniy Kirichenko, Vladimir Sobolev, Ksenia Kirichenko

**Affiliations:** 1 Membrane Institute, Kuban State University, 149 Stavropolskaya st., 350040 Krasnodar, Russia; 2 Frumkin Institute of Physical Chemistry and Electrochemistry RAS, 31 Leninsky prospect, 119071 Moscow, Russia; 3 Kuban State Agrarian University named after I.T. Trubilin, 13 Kalinina st., 350004 Krasnodar, Russia

**Keywords:** electrodialysis, ion exchange membrane, MK-40, MF-4SC, polyethyleneimine, membrane modification, polyelectrolyte coating, zeta potential, current-voltage curve

## Abstract

Coating of ion exchange membranes used in electrodialysis with layers of polyelectrolytes is a proven approach that allows for the increasing of the limiting current, the suppressing of sedimentation, the controlling of the intensity of generation of H^+^ and OH^−^ ions, and also the improving of monovalent selectivity. However, in the case when two materials with the opposite sign of the charge of fixed groups come in contact, a bipolar boundary is created that can cause undesirable changes in the membrane properties. In this work, we used a MK-40 heterogeneous membrane on the surface of which a layer of polyethyleneimine was applied by adsorption from a solution as a model of heterogeneous membranes modified with oppositely charged polyelectrolyte. It was found that, on one hand, the properties of modified membrane were beneficial for electrodialysis, its limiting current did not decrease and the membrane even acquired a barrier to non-selective electrolyte transport. At the same time, the generation of H^+^ and OH^−^ ions of low intensity arose, even in underlimiting current modes. It was also shown that despite the presence of a layer of polyethyleneimine, the surface charge of the modified membrane remained negative, which we associate with low protonation of polyethyleneimine at neutral pH.

## 1. Introduction

Electrodialysis is a method of desalination, concentration, and fractionation of solutions based on the selective transport of ions through charged membranes under the action of an external electric field [1]. Electrodialysis with ion exchange membranes is traditionally used in water treatment [2], production of acids and bases [3,4], recycling of technological solutions [5], correction of food composition [6,7], and table salt production [8]. In the simplest case, electrodialysis purification means the removal of any ions, i.e., a decrease in the total salinity [1]. This is true for production of potable or deionized water [9,10] and water for the food industry [6,11]. However, there are also applications in which the removal of ions of a certain type is important. For example, it is necessary to remove Mg^2+^ and SO_4_^2−^ and preserve mainly NaCl to concentrate table salt from sea water [12]. It is also required to remove Ca^2+^ and SO_4_^2−^ ions, the simultaneous presence of which leads to the precipitation of gypsum when treating such complex mixtures as mining wastewaters [13]. For central heating services, Mg^2+^, Ca^2+^, HCO_3_^−^, and CO_3_^2−^ ions are removed, as they can cause scale formation [14]. To achieve tartrate stability of wines, anions of tartaric acid are removed [15], whereas potassium is preferably allowed to remain for its health benefits, and moreover, in the dairy industry, NaCl and KCl are removed from milk whey [16], whereas calcium and organic ions may remain.

As can be seen from the aforementioned examples, some of the tasks of selective separation can be reduced to the problem of separating doubly charged ions from their mixtures with mono-charged ions. In electrodialysis, there is no internal mechanism for ensuring selectivity by charge number, which exists, for example, in ultrafiltration and nanofiltration [17]. It is achieved through creation of special grade-selective membranes. Earlier, in order to increase monovalent selectivity, proposals were made to increase the hydrophobicity of membranes [18], to apply a crosslinked or self-crosslinking polymer that would create a size-exclusion network [19,20] or to apply a layer of a substance carrying the dissociating groups of the same sign of charge as the removed ions for increased electrostatic repulsion [21]. These techniques allowed for the achievement of a rather modest increase in selectivity [22], yet, depending on the approach, the occurrence of undesirable effects such as a decrease in electrical conductivity and a generation of H^+^ and OH^−^ ions were reported. A novel widely used approach in creating membranes with increased monovalent selectivity is the deposition of polyelectrolyte layers with an alternating charge sign of fixed groups [23,24,25,26,27]. For a system with such membranes, White et al. achieved a K^+^/Mg^2+^ selectivity over 1000 with a moderate increase in electrical resistance [28].

A significant advantage of the layer-by-layer approach is the possibility of production of selective membranes on the basis of a wide range of supports—employed membranes vary from very expensive Nafion ion exchange membrane [23] to filtration membranes [24], and creation of supportless layers has also been reported [29].

For the case of heterogeneous bipolar membranes used as supports, however, in our previous work [30] it was shown that application of even a relatively thin layer of polyelectrolyte carrying the functional groups charged oppositely to fixed groups in membrane bulk significantly reduced the limiting current density, whereas it was far less pronounced for homogeneous membrane. At the same time it is known that application of homogenizing layer at the surface of heterogeneous membrane reduces the concentration polarizations and boosts the limiting current density [31]. This allowed us to propose the following approach to the creation of selective membranes based on a heterogeneous membrane:

First, a cheap heterogeneous membrane was chosen as a substrate. We picked standard grade MK-40 cation exchange membrane manufactured by Shchekinoazot, Russia [32], that, according to manufacturer data and independent studies [33], is characterized by high mechanical and chemical stability and high counterion transport numbers. The disadvantages of this membrane are high electrical resistance caused by the polyethylene content exceeding 30% and the low limiting current of salt counterions, which is about 0.7 of theoretical limiting current calculated by the Lévêque equation [31]. The decrease in the limiting current is caused by a higher density of electric current lines in the conducting sections as compared with a homogeneous membrane, which leads to an increase in the local concentration polarization [34].

Second, an ion exchange membrane was coated with a thin layer of a polyelectrolyte carrying the functional groups of the same sign of charge as functional groups in membrane bulk. This modification was made earlier for MK-40 and Nafion dispersion in [31,35], where the authors attribute the observed improvement in properties to a more uniform distribution of electric current streamlines along the membrane surface and to increased hydrophobicity, which facilitates the movement of convective vortices within a solution near the membrane surface. As a result, the properties of the modified heterogeneous membrane approach the properties of the Nafion homogeneous membrane, and an economical benefit can be gained from the difference in the cost of materials.

Third, polyelectrolytes, the sign of the charges of the fixed groups of which will alternate from layer to layer, were applied on the existing layer of cation exchange polyelectrolytes. According to Abdu et al. [36], in the case when the fixed groups of the upper modifying layer (that contacts with the solution) are charged oppositely to the fixed groups of the membrane bulk, the modified sample will be characterized by increased monovalent selectivity.

In this work evaluated the possibilities of the proposed approach using as a model a heterogeneous membrane modified with only two layers—a homogenizing layer and a polyelectrolyte layer carrying functional groups that are oppositely charged with respect to the functional groups of the membrane bulk. For evaluation of effect of modification on operation of membrane in electrodialysis, the ways in which coating with polyelectrolyte layers changes the thickness and relief of the membrane, its electrical conductivity and diffusion permeability, zeta potential, and limiting current density value determined from current–voltage curves (CVC) were determined.

## 2. Materials and Methods

### 2.1. Solutions

Solid NaCl and solid CaCl_2_, both analytic grade, and 99% isopropyl alcohol were bought from Vekton JSC, Russia. Distilled water was produced on site. Polyelectrolytes are described in Section 2.2. All measurements were performed at 25 °C, with the exception of the registration of steaming potentials and currents that were carried out at 20 °C.

### 2.2. Membranes and Modifiers

We used the Russian commercially available MK-40 heterogeneous cation exchange membrane manufactured by Shchekinoazot [32]. Shchekinoazot reports the production as follows: a mixture of powdered polyethylene (grain diameter less than 5 microns), sulfonated styrene-divinylbenzene KU-2 resin (grain diameter less than 50 microns), and antioxidants [33] are hot rolled between two reinforcing cloths made of Nylon 6. A feature of the resulting membrane is the presence of a continuous phase of polyethylene both on the surface and in the bulk of the membrane. Polyethylene makes 30%–40% of the membrane bulk, but the surface fraction of polyethylene is higher; on the basis of scanning electron microscopy, different authors estimate it as 75%–81% [37] and 83%–87% [38]. Between the ion exchange grains and polyethylene, there are gaps forming macropores, with their diameter being about 1–10 microns [39].

Shchekinoazot also produces other membranes by the same technology. MA-41 anion exchange membrane differs from MK-40 cation exchange membrane mainly by functional groups—instead of sulfonic groups, its styrene-divinylbenzene ion exchanger bears trimethylammonium groups. These quaternary ammonium bases may undergo transformation into different types of amines and may even be totally eliminated due to passage of electric current and strong alkalinity of contacting solution [40], and thus the functional groups of operating membrane are not 100% quaternary ammonium bases. In our previous study [30], we coated MA-41 series membranes with Nafion and found that the resulting membrane had significantly reduced limiting current of salt counterions.

Membranes were prepared for the study by conversion to the Na^+^ form as follows. First, the sample was placed in 96% ethanol for 1 h, then it was transferred to a saturated (32% at room temperature) NaCl solution for 1 additional hour, after which the solution was diluted twofold, the dilution repeated six times with an interval of 1 hour, then the sample was placed in a NaCl solution of the concentration required in the experiment for which the samples were intended, and the solution was periodically changed until its conductivity was constant. This method is based on salt treatment described in [41]. To conduct experiments with samples in the Ca^2+^ form, pretreated samples in the Na^+^ form were placed in a CaCl_2_ solution of the required concentration, the solution was periodically replaced until its electrical conductivity became constant. The steps starting from the sample being placed in a saturated solution and ending in the electrical conductivity of equilibrating solution becoming constant are referred below as “stepwise equilibration”, after which it is indicated with which solution the equilibration was made.

**Figure 1:**
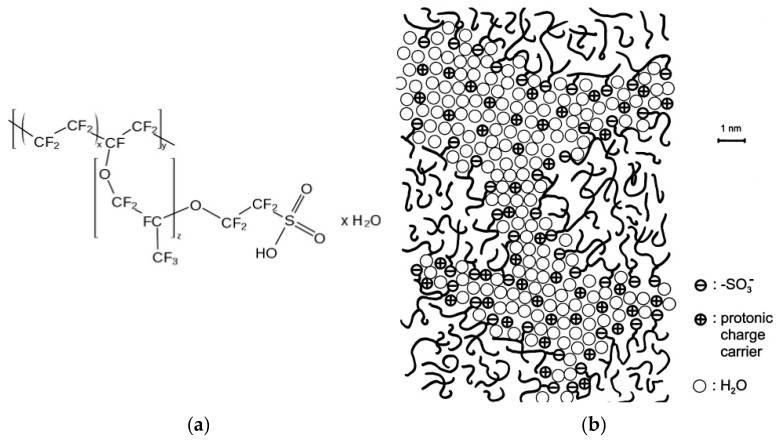
The generalized chemical formula of Nafion (**a**) and the model of its structure proposed by Kreuer, reprinted with permission from [44]. Copyright (2001) Elsevier Science B.V. (**b**).

The perfluorinated cation exchanger containing sulfonic groups used as a homogenizing material was manufactured by the Plastpolymer JSC under the MF-4SC trademark. It is the Russian analogue of the Nafion material [42], and the Nafion structural formula and the model for structuring the polymer in a swollen state shown in Figure 1 are applicable to it. For the modification, 20 wt % dispersion of MF-4SC in lower aliphatic alcohols and water diluted to 7% with isopropyl alcohol was used. When drying on the surface of a heterogeneous membrane, a dispersion of similar concentration formed continuous homogeneous films, as shown in [30,43].

**Figure 2:**
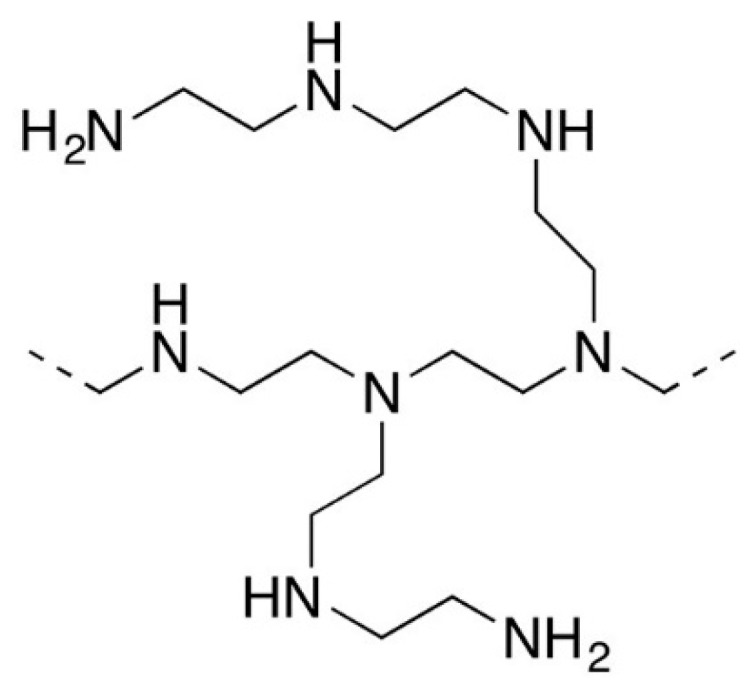
Structure of polyethyleneimine (PEI) unit.

Polyethyleneimine (PEI), used as an anion exchange polyelectrolyte in this work, can be linear or branched. In our case, a branched variety with an average molecular weight of 10,000 manufactured by Sigma-Aldrich was used. The structure of the polymer unit is shown in Figure 2.

As can be seen from the figure, this is an aliphatic polymer containing primary, secondary, and tertiary amino groups. They are able to enter the electrostatic interaction with sulfonic groups of Nafion or MF-4SC, and due to this PEI bonds with the membrane surface.

The modifier was purchased in solid form, and before use it was dissolved in isopropyl alcohol to produce a solution with a concentration of roughly 1 g/L (precisely 1.1928 g/L).

### 2.3. Modification Technique

MK-40 membrane equilibrated with 0.02 M NaCl solution was cut into 6 × 6 cm^2^ pieces. Some of these pieces were left nonmodified for comparison; this nonmodified series is denoted below as MK-40, whereas others were modified. At the first step of the modification, the samples were dried at 60 °C in a Binder FD 115 drying and heating chamber for 1 hour and then cooled to 25 °C, and then were fixed with an adhesive tape to the bottom of a Petri dish. The dish was washed with isopropyl alcohol and dried beforehand. The duct tape was glued to the outer part of the membrane, leaving a window in the center for the modification; the area of the window was 3.5 × 3.5 cm^2^. Then, the working window was degreased by rubbing with isopropyl alcohol. After that, 0.2 ml of the 7% dispersion of MF-4SC was spread on the surface of the operating window using the spatula, and the sample was left at room temperature until the solvent evaporated. Then, samples were heated for 1 hour at 50 °C, and after that they cooled to room temperature. Some of these membranes, denoted below as MK-40+1, were then unattached from the adhesive tape, stepwise equilibrated with 0.5 M NaCl solution or 0.25 M CaCl_2_ solution, and used for conductometry or diffusion permeability measurement as a comparison. For the remaining membranes, 50 mL of 1.1928 g/L PEI solution in isopropyl alcohol was poured into the Petri dish and kept for 1 h for the modifier to adsorb onto the membrane surface. After that, the solution was discarded, the membrane was dried for 1 hour at 50 °C, cooled at room temperature for 24 hours, and stepwise equilibrated with a 0.02 M NaCl solution. These samples are denoted as MK-40+2.

The thermal treatment of the modifying layers was due to the fact that in preliminary experiments we found that an increase in temperature during the coating of ion exchange membrane with polyelectrolyte in otherwise equal conditions led to the formation of a thinner, i.e., denser, polymer layer, which seemed promising for the creation of a barrier layer.

### 2.4. Thickness Measurement

The membrane thickness was measured with an MKC-25 0.001 micrometer (Micron Ltd., Moscow, Russia). Its nominal accuracy was 0.1 microns. For each sample, the thickness was obtained as the average of 10 measurements at points relatively uniformly distributed over the entire membrane area.

Thicknesses were determined for swollen samples stepwise equilibrated with 0.5 M NaCl or with 0.25 M CaCl_2_ solutions. Immediately before the measurement, a membrane was removed from the solution, nonmodified areas were wiped with filter paper and modified areas were dried by air blast, thickness was quickly determined, and then the sample was returned to the solution.

### 2.5. Atomic Force Microscopy

To confirm the success of the modification and determine the character of the distribution of polyelectrolytes, atomic force microscopy was used in contact mode using a Jeol 5400 microscope (JEOL Ltd., Akishima, Japan) of the Diagnostics of the Structure and Properties of Nanomaterials Center for Collective Use of the Kuban State University. Samples were initially stepwise equilibrated with 0.02 M NaCl solution, but before atomic force microscopy they were dried and then examined in a dry state. The topography measurements were repeated five times for different parts of the surface of one sample from each series of membranes. As a result, five sets of visualizations of the surface topography and of roughness parameters were obtained. Two roughness parameters were selected for comparison: the arithmetic average of the roughness profile, *R_a_*, and the ratio of the true area of the membrane to the ideal (absolutely flat) area, *S*_ratio_.

### 2.6. Electrical Conductivity Measurements

**Figure 3:**
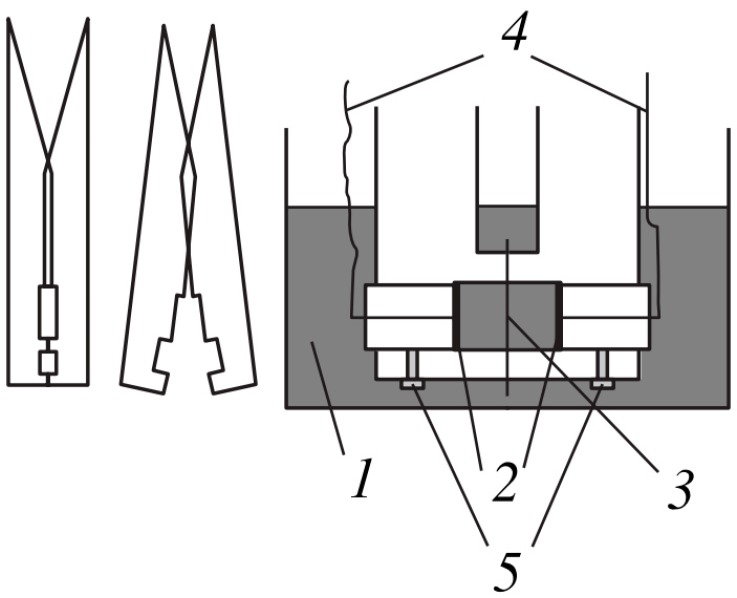
The clip cell for determining the electrical conductivity of the membranes by the difference method (reprinted with permission from [46]. Copyright (2001) MAIK “Nauka/Interperiodica”.). 1—vessel with equilibrium solution, 2—Pt/Pt electrodes, 3—membrane, 4—insulated conductors, 5—screws fixing position of electrodes.

The electrical conductivity of the membranes was determined at alternating current by the difference method using clip cell (Figure 3); details of the methodology are given in [45].

Important limitations of the different method origins from the procedure when the electrical resistances of the membrane in solution and of solution without a membrane were measured, and then the membrane conductivity was calculated by the Equation [45]. At low solution concentrations, including the concentration 0.02 meg/L used for registration of the CVC and streaming potentials, the solution resistance is very high compared to the membrane resistance; hence, the resistance of membrane in solution, *R_m+s_*, becomes approximately equal to the solution resistance, *R_s_*. As a result, the denominator of the fraction in Equation (1) becomes a small value that possesses the measurement uncertainty, and the electrical conductivity is determined with a large uncertainty. For this reason, to assess the change in electrical conductivity as a result of applying the modifying layers, the samples were measured equilibrated with either 0.5 M NaCl or 0.25 M CaCl_2_ (which corresponds to 0.5 meq/L CaCl_2_). In addition, many sources, including, for example, sites of membrane manufacturers [32,47], give the conductivity of membranes in 0.5 M solutions, and thus this concentration is more general for comparison.

(1) $$\kappa_{m}=\frac{d_{m}}{R_{m+s}-R_{s}}$$

where *κ_m_* is membrane conductivity, *d_m_* is membrane thickness, *R_m+s_* is the resistance of membrane in solution, and *R_s_* is the resistance of solution without membrane.

Electrical conductivity measurements were repeated five times at different points of the sample.

### 2.7. Streaming Potential Measurements: Calculation of Zeta Potential and Surface Charge

**Figure 4:**
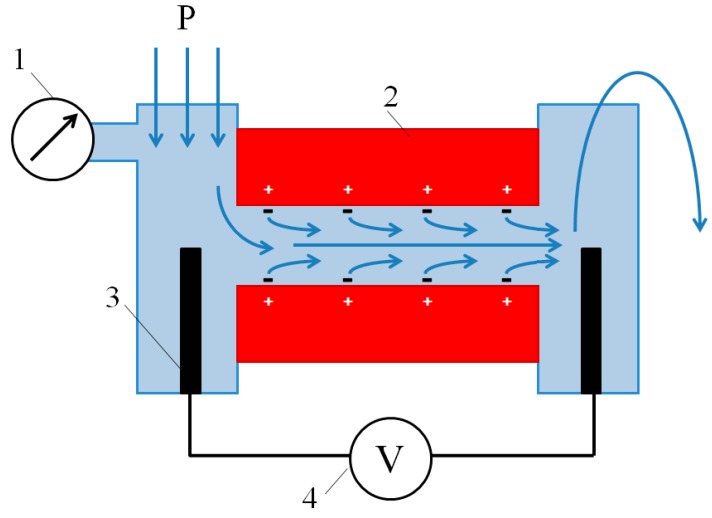
Scheme of the gap cell for measurement of streaming potential or streaming current. The scheme depicts the case of measurement of streaming potential of a cation exchange membrane. 1—pressure gauge, 2—studied samples, 3—Ag/AgCl electrode, 4—multimeter. Blue arrows show the direction of liquid flow from inlet (three arrows at left side) to discharge (curved arrow at right side). Reprinted with permission from [51]. Copyright (2017) Elsevier Ltd.

To calculate the surface charge, σ, we used the value of the zeta potential, ζ, which, in turn, was determined from the experimental data of measurements of the streaming potential. The gap cell used for measurement of streaming potential and streaming current of ion exchange membranes is described in [48], and is schematically shown in Figure 4. It is similar to the cell applied in the Anton Paar SurPass 3 electrokinetic analyzer. The latter was employed by Yaroshchuk and Luxbacher [49] for determination of external and internal (inside membrane pores) streaming potentials, as well as by Sedkaoui et al. [50], who developed a promising method for finding the lateral conductivity of ion exchange membranes from the value of the streaming current and streaming potential. In our cell, two samples under study formed a slit parallelepipedal channel 25 mm long, approximately 2 mm (from 1.8 to 2.5 mm, depending on the experimental run) wide, and 20–70 μm high. The cell was placed in the N_2_ atmosphere with an overpressure so that the cell itself and the solution at its inlet were under controlled overpressure, and the outlet from the cell was open to the atmosphere. The generated overpressure varied in the range 0.125–2.000 bar. Streaming potentials and streaming currents were registered with the help of two open Ag/AgCl electrodes (Figure 4) using a RIGOL DM3058E multimeter (RIGOL Technology Co., Ltd., Suzhou, China). The multimeter can be connected to run measurements both in voltmeter mode to determine the streaming potentials and in ammeter mode to determine the streaming currents.

To determine the effect of electrolytes, we tested samples in NaCl and CaCl_2_ solutions. To find the effect of desalination occurring during electrodialysis on zeta potentials, we studied the streaming potentials and streaming currents both in the 0.02 meg/L solutions, that is, concentration used for CVC registration, and in 0.002 meg/L (diluted 10-fold) solutions. In all cases, experiments were conducted at 20 °C.

When the zeta potential is calculated from the experimental dependence of the streaming potential on the pressure difference between the outlet and inlet of the cell ΔE/Δp, the classical Helmholtz–Smoluchowski equation is the most often used [52,53,54,55]:

(2) $$\zeta =\frac{\Delta E}{\Delta p}\frac{\eta \kappa_{0}}{\varepsilon \varepsilon_{0}},$$

where *η* is the dynamic viscosity coefficient, *κ*_0_ is the conductivity of the solution feeding the gap cell, *ε* is the relative permittivity of solution, and *ε*_0_ = 8.85 × 10^−12^ F/m is the vacuum permittivity.

However, this equation was deduced for the channel with nonconductive walls. For the first time, the account of electrical conductivity of the walls was made by Yaroshchuk and Ribitsch [56] and then applied by Yaroshchuk and Luxbacher [49], Fievet et al. [57], and Szymczyk et al. [49,50] for determination of zeta potential, and by Sedkaoui et al. [50] for finding membrane lateral conductivity. Starting from the Yaroshchuk and Ribitsch equation, in one of our previous works [51], we deduced the equation that takes into account not only electrical conductivity, but also roughness of the channel walls:

(3) $$\zeta =\frac{\Delta E}{\Delta p}\frac{\eta \kappa_{0}}{\varepsilon \varepsilon_{0}\gamma }\left(1+2\frac{\kappa_{m}d_{m}}{\kappa_{0}h}\right),$$

where *κ_m_* is the membrane conductivity, *d*_m_ is the membrane thickness, *h* is the intermembrane distance (in our case it is the channel height), and γ is the ratio of surface profile length to the length of baseline. The ratio of surface profile length to baseline appears when calculating the streaming current by integrating the local charge density and fluid velocity and then taking into account that, at the same surface concentration there, are more charges located near the rough surface than near the smooth surface. Assuming that the membrane surface is isotropically rough, the ratio of the surface profile length to the base length can be calculated from known from atomic force microscopy ratio of the true area of the membrane to the ideal (absolutely flat) area *S_ratio_*:

(4) $$\gamma =\sqrt{S_{ratio}}.$$

Note that when the membrane does not conduct electric current or its conductivity can be neglect+ed in comparison with the conductivity of the solution, i.e., when $\frac{\kappa_{m}d_{m}}{\kappa_{0}h}$ <<1, and its surface is ideally smooth, i.e., γ = 1, Equation (3) is reduced to the Helmholtz–Smoluchowski equation (Equation (2)).

Because a direct determination of the membrane conductivity in 0.1 M or more diluted solutions using the difference method and the clip cell is impossible for the reasons described in Section 2.6, we acted as follows. For each experimental run, the electrical resistance of the gap cell was determined from registered streaming potential and streaming current; then, the geometric parameters of the channel were measured at the disassembled cell, and following this the effective electrical conductivity of the channel was calculated, which, in turn, was substituted into the Helmholtz–Smoluchowski equation (Equation (2)). The resulting equation has the following form:

(5) $$\zeta =\frac{\Delta E}{\Delta p}\frac{\eta I_{s}L}{\varepsilon \varepsilon_{0}Ehb},$$

where *E* and *I_s_* are the streaming potential and the streaming current, respectively, registered for the same value of pressure drop; *L* is the channel length; *h* is the intermembrane distance; and *b* is the channel width.

By analogy with Equations (2) and (3), Equation (5) can be modified to include the roughness of surface as follows:

(6) $$\zeta_{N}=\frac{\Delta E}{\Delta p}\frac{\eta I_{s}L}{\gamma \varepsilon \varepsilon_{0}Ehb}.$$

The surface charge can be calculated from known zeta potential by the Grahame equation [58]:

(7) $$\sigma =\sqrt{8\varepsilon \varepsilon_{0}CRT}\times \sinh\left(\frac{\zeta F}{2RT}\right),$$

where *C* is the electrolyte concentration, *R* = 8.314 J/(mol K) is the gas constant, *T* is the absolute temperature, and *F* = 96485 C/mol is the Faraday constant.

### 2.8. Current–Voltage Curves

**Figure 5:**
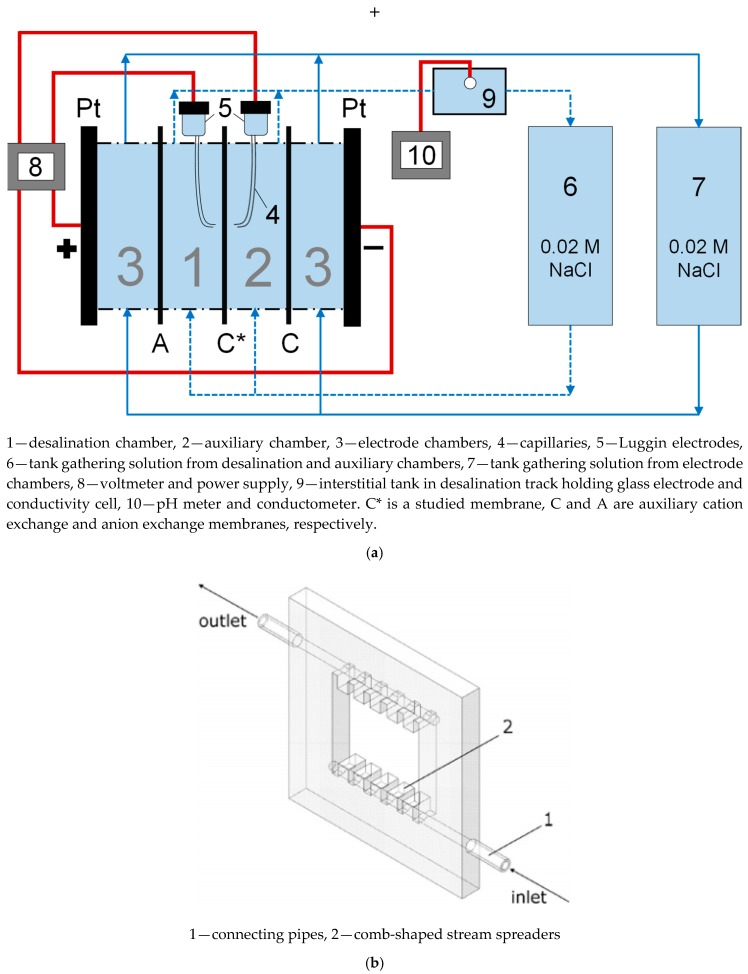
Four-chamber flow-through cell used to register the current–voltage curves (CVCs) (**a**), adapted with heavy edits from [30], and the frame (**b**) forming the walls of one chamber (in this case, the electrode chamber), reprinted with permission from [59]. Copyright (2011) American Chemical Society.

To register the CVCs, we used the laboratory cell described in [30]; its principal scheme is given in Figure 5.

The studied sample and two auxiliary membranes (MK-40 cation exchange and MA-41 anion exchange heterogeneous membranes manufactured by Shchekinoazot) were separating four flow chambers—a desalination chamber, an auxiliary chamber, and two electrode chambers. Modified membranes were placed in such a way that the modifying layer faced the desalination chamber and substrate membrane faced the auxiliary chamber, i.e., the modifying layer was closer to the anode, whereas the cation exchange membrane was closer to the cathode. Such orientation was chosen for possibility of easier comparison with the studies of monovalent selectivity [23,36]. There are also studies showing that the shape of CVC of asymmetrically modified membranes may change depending on the membrane orientation [60], and it was shown that the membrane orientation employed in our study would produce lower limiting current densities than the opposite orientation.

The total membrane area was 6 × 6 cm^2^, but only the central 2.15 (length) × 2 (width) cm^2^ window was polarized (made contact with the solution and was affected by the external electric field). The solution was supplied to all chambers by gravity force from containers located above the cell. The treated solution was returned to the circulation tanks by the Heidolph Hei-FLOW Precision 01 multichannel peristaltic pump (Heidolph Instruments GmbH & CO. KG, Schwabach, Denmark). The first circulation tank collected the solution from desalination and auxiliary chambers, and the second tank from the electrode chambers. In these experiments, we controlled the pH value, and in each case the starting pH was about 6–7. To ensure the laminarity of the flow within the cell, plastic frames with spreaders were used (Figure 5b); the shape and location of spreaders were calculated earlier and the frames were widely reported in previous works of our laboratory [37,59]. The intermembrane distance was 0.59 cm. The limiting current density for such geometric parameters can be calculated using the Lévêque equation [61]:

(8) jlimtheor=z1C1FDh(T1−t1)[1.47(h2V0LD)13−0.2],

where *z*_1_ and *C*_1_ are the charge and the molar concentration of counterion in solution bulk, respectively; *D* is the diffusion coefficient of salt in solution; *h* is the intermembrane distance; *T_1_* and *t_1_* are the counterion transport number in membrane and in solution, respectively; *V*_0_ is the linear solution pumping rate; and *L* is the length of desalination path. *D* value is taken to be equal to that at infinite dilution and 25 °C, which is 1.61 × 10^−9^ m^2^/s for NaCl and 1.335 × 10^−9^ m^2^/s for CaCl_2_ [62]. For the experimental conditions, *T_1_* was assumed to be equal to 1 (as the membranes are highly selective for counterions in such dilute solutions, and at currents close to the limiting current density the input of water splitting was found to be negligible), *t*_1_ was 0.603 for Na^+^ in NaCl and 0.438 for Ca^2+^ in CaCl_2_, *h* was 0.59 cm, and *L* was 2.15 cm. The calculated limiting current density was 1.96 mA/cm^2^ for NaCl and 1.86 mA/cm^2^ for CaCl_2._

The Lévêque equation for the limiting current density is applicable only for a single-layer membrane. Filippov et al. [60,63] studied the asymmetry of the limiting current densities of bilayer membranes depending on their location in the measuring cell and deduced the implicit algebraic formulas for calculation of the values of the limiting current densities from the data extracted from diffusion measurements in two orientations of the membrane. Comparison of the data obtained for different orientations of the membrane may give additional insight on its structure but is currently beyond the scope of our study. In this work, we used the Lévêque equation for calculation of the theoretical limiting current densities as a means of comparison with a single layer membrane.

The cell had two polarizing platinum and two measuring Ag/AgCl electrodes connected to a Autolab PGStat N100 power source voltmeter (Metrohm AG, Herisau, Switzerland). Ag/AgCl electrodes were connected to Luggin capillaries glued into the frames forming desalination and auxiliary chambers in such a way that the tips of the capillaries were projected onto the geometric center of the membrane and were opposite each other on different sides of the membrane at a distance of about 0.15 cm from its surface. This distance was much greater than the thickness of the diffusion layer calculated for this system by the equation [64] obtained when combining the Peers [65] and the Lévêque equations, which was, in the cases of both 0.02 M NaCl solution and 0.01 M CaCl_2_ solution, about 0.025 cm. This equation for the calculation of the average thickness of diffusion layer, δN_av_, is written as follows:

(9) δN av=1.02h(LDh2V0)13.

The measurements were carried out in galvanodynamic mode with a linear sweep of the current density from 0 to 5 mA/cm^2^ at a rate of 2.5 × 10^−3^ mA/(cm^2^s). For each membrane, the measurements were repeated twice and the obtained CVCs were found to be matching.

There was an interstitial tank at the outlet of the desalination chamber, in which, as well as in all circulation tanks, a conductometric cell was mounted, connected to an Expert 002 conductometer, and a combined glass electrode was, mounted connected to an Expert 001 pH meter (both produced by Econix-Expert Ltd., Rumyantsevo, Russia).

### 2.9. Diffusion Permeability

To measure diffusion permeability, we used a two-chamber flow-through cell, similar in design to the cell for registration of CVC. Its chambers were formed by the studied membrane, by organic glass frames that possessed solution input and output devices, and by tie plates. The total area of the used membranes was 5 × 5 cm^2^ (samples were cut to fit), whereas only a 2 × 2 cm^2^ central window was available for mass transfer. As with CVC, in diffusion experiments, changing orientation of a bilayer membrane may result in different values of fluxes and permeability [66]. Obtained values may be analyzed to calculate the coefficients of diffusion and ionic equilibria in a layer, which then may be used to calculate the CVC. However, as with CVC, in this work we focused on the case in which the membranes were also placed in such a way that the modified surface was the first to face the ionic flux, i.e., modifying layers faced the “salt” chamber, and the nonmodified side of the supporting membrane faced the “water” chamber.

Each chamber was connected by pipes with its own tank; in this way, upstream and downstream tracts were formed, with the volume of solution in each being 400 mL. The upstream tract was filled with a salt solution, and the downstream tract was filled with distilled water. Thus, according to Fick’s first law [67], the flux of salt, and, accordingly, the greater the rate of growth of concentration of salt in the downstream tract, the higher the concentration of salt in the upstream tract. In our case, to create the salt flux necessary to complete the experiment within a reasonable time, 0.5 M NaCl was used to study the diffusion of NaCl and 0.25 M CaCl_2_ was used to study the diffusion of CaCl_2_. The increase in electrolyte concentration was calculated by the change in conductivity detected by the conductivity cell located in the receiving tank and connected to the Expert 002 conductivity meter; the conversion factor for each electrolyte was determined in advance. It was assumed that a decrease in the concentration of electrolyte in the upstream tract during the experiment can be neglected.

## 3. Results and Discussion

### 3.1. Thickness and Roughness

**Figure 6:**
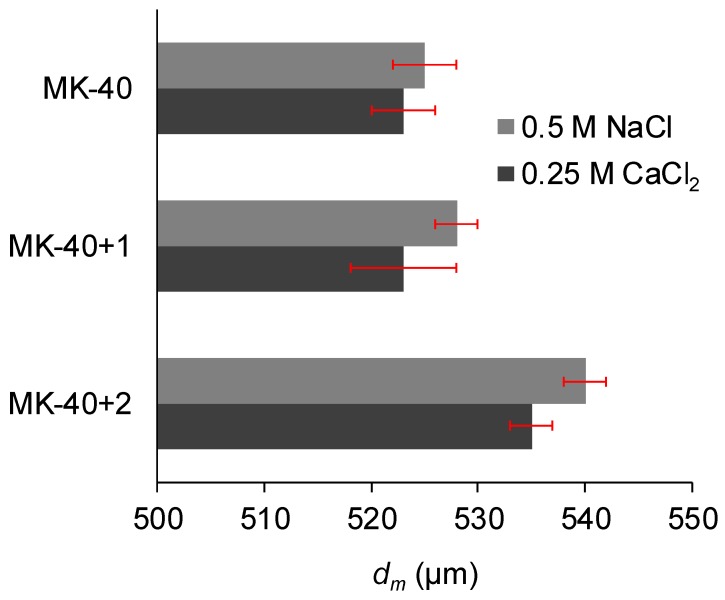
The thickness of the commercial MK-40 membrane and the modified membranes based on it. Light gray indicates the thickness of the membranes equilibrated with 0.5 M NaCl, whereas dark gray indicates the thickness of the membranes equilibrated with 0.25 M CaCl_2_. Margins of error show the confidence interval calculated for α = 0.05.

**Figure 7:**
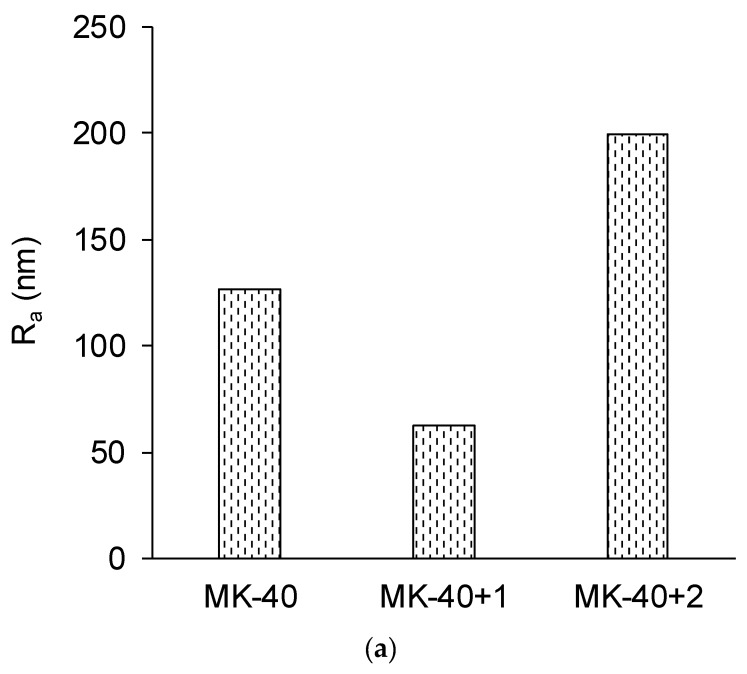

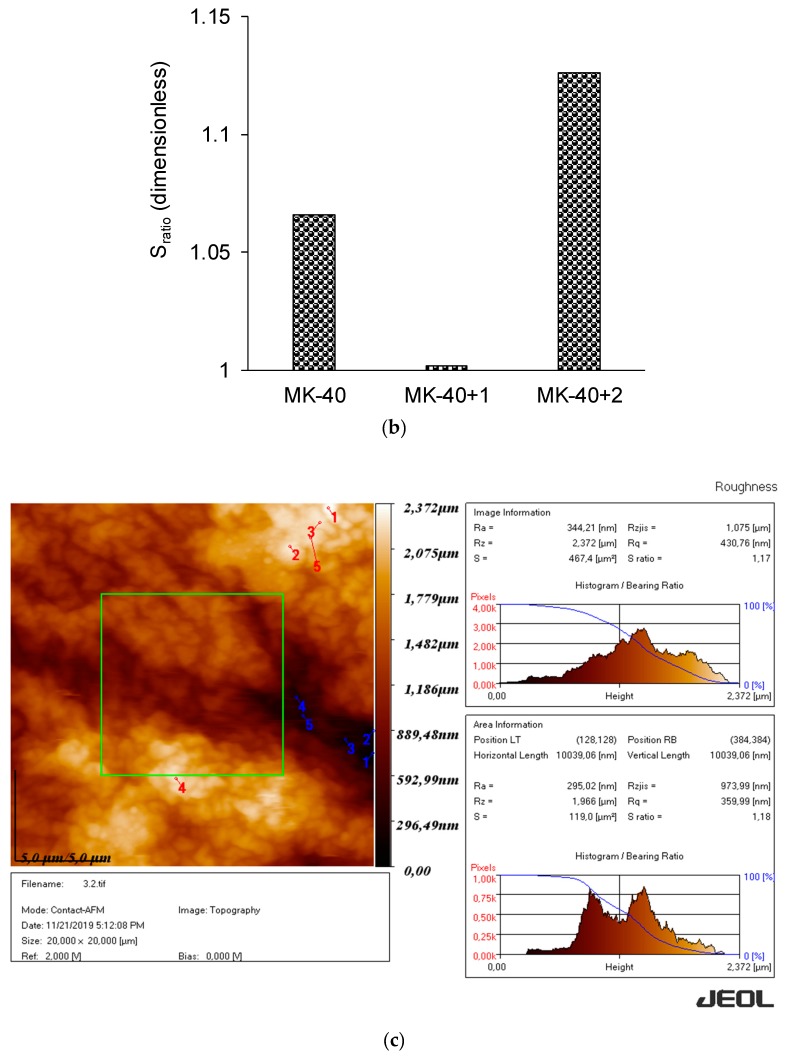
The roughness profile, *R_a_* (**a**), and the ratio of the true area of the membrane to the ideal (absolutely flat) area, *S*_ratio_ (**b**) roughness parameters of the membranes, determined using atomic force microscopy, and an example of the obtained visualization of surface profile of the MK-40+2 membrane (**c**). Other profiles are given in the Supplementary Materials.

Figure 6 shows the membrane thicknesses measured with a micrometer, and Figure 7 shows the roughness parameters: the arithmetic average of the roughness profile, *R_a_*, and the ratio of the true area to the base area, *S_ratio_*. An increase in membrane thickness after the application of each modifying layer was expected, but MF-4SC layer was found to be thin, whereas the significant thickness of the PEI layer was worth noting.

A decrease in the roughness of a heterogeneous membrane after applying a homogenizing layer was expected and can be attributed to the filling of surface defects with polyelectrolyte. The increase in both roughness parameters after applying the second modifying layer was unexpected. The exact reason for this is not clear, but the shape of the inhomogeneities (Figure 7c) allows us to suppose that they were formed by polymer chains that, due to the complex branched chemical structure, became entangled.

### 3.2. Surface Charge and Zeta Potential

Table 1 lists the experimental dependences of the streaming potential on the overpressure, the zeta potentials ζ calculated by Equation (5) that did not take into account the total membrane area being larger than the area of ideally flat membrane, the zeta potentials ζ_N_ calculated by Equation (6) that took into account the larger membrane area, and the corresponding surface charges σ and σ_N_ calculated by the Grahame equation (Equation (7)).

It can be seen that the commercial MK-40 cation exchange membrane and its modification with a layer carrying amino groups were same charged to each other and oppositely charged to the previously studied AMX-Sb anion exchange membrane [51]. Several conclusions can be drawn from this.

First, it means that the applied PEI layer was either negatively charged, which was unlikely given that it contained amino groups and that, if it were specifically adsorbing the Cl^–^, it would be noticed in earlier studies, or that the layer used by us was insufficient to compensate for the charge of sulfonic groups of the membrane substrate and the underlying modifier layer. This may have happened either because of the small amount of applied PEI, which was unlikely given that, according to the measurements, the thickness of this layer was about 10 μm, or because in the neutral pH range, in which the experiments were carried out, most of the amino groups were deprotonated and did not contribute to the formation of a surface charge. The second hypothesis is supported by published data, according to which [68], in the neutral pH range, about 80% of the nitrogen atoms that are present in the PEI are deprotonated. These data are of great importance for the creation of monovalent selective membranes, as PEI is a popular modifier, and the selectivity of modified samples substantially depends on the surface charge [69]. The disagreement with studies that shows that in layer-by-layer systems the PEI layer is positively charged might be due to the fact that, in thin layers, more nitrogen in PEI is deprotonated due to proximity to strongly acidic groups of other layers, or that cation exchange groups in layers described in earlier works are present in a smaller amount than in the membrane used in this work, and hence their charge is easier to compensate; however, this question requires further testing.

Second, no change in sign of charge meant the absence of specific sorption of Ca^2+^ ions at any membrane, which would be a mechanism of growth of diffusion permeability if such a change were present.

Finally, because the membranes always carried a negative charge, the electric double layer in the solution would have been formed by cations.

Aside from constant sign of charge, it can be seen that upon the transition from NaCl to CaCl_2_, the absolute values of zeta potential and of surface charge decreased, meaning that Ca^2+^ ions were more effective in screening the charge of fixed sulfonic groups.

A decrease in absolute value of the surface charge was also observed, with increasing concentration of the equilibrium solution. This is consistent with reports of the loss of monovalent selectivity with growing ionic strength [24]. On the other hand, this meant that with a decreasing concentration of solution occurring during desalination, the (absolute value of) surface charge would increase, and because growth of the surface charge enhances electroconvection [51], then the increase in (absolute value of) charge during desalination should be considered as another factor that boosts the mass transport during electrodialysis desalination.

### 3.3. Electrical Conductivity and Diffusion Permeability

**Figure 8:**
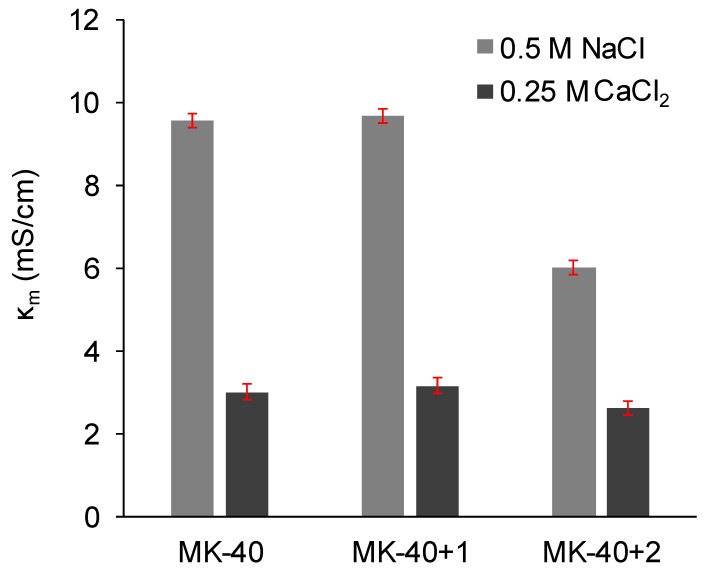
The electrical conductivity of the commercial MK-40 membrane and modified membranes based on it. Light gray indicates the conductivity of the membranes equilibrated with 0.5 M NaCl, dark gray indicates the conductivity of the membranes equilibrated with 0.25 M CaCl_2_. Margins of error show confidence interval calculated for α = 0.05.

Figure 8 shows a comparison of the membrane conductivities in a 0.5 M NaCl solution and in a 0.25 M CaCl_2_ solution. In all cases, the electrical conductivity in 1:1 electrolyte was found to be higher than in 2:1 electrolyte. Application of a MF-4SC layer did not change the electrical conductivity, but the application of a PEI layer decreased the electrical conductivity, only marginally in the case of NaCl solution but quite significantly in the case of CaCl_2_ solution.

**Figure 9:**
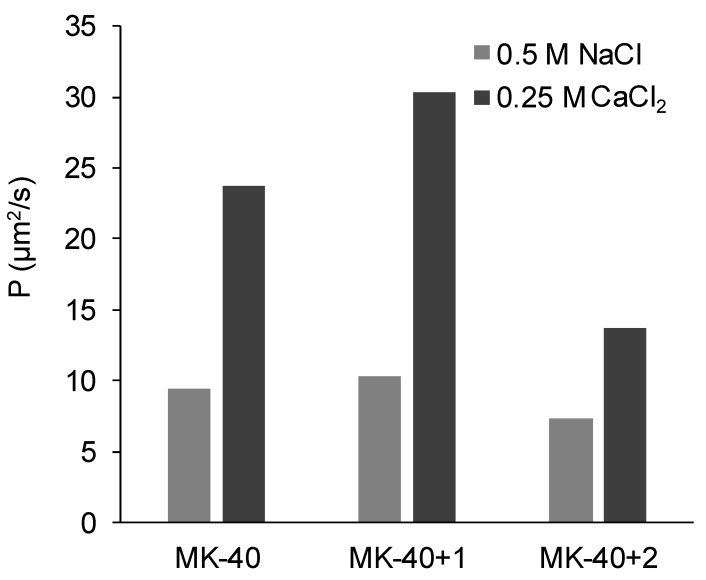
The diffusion permeability of the commercial MK-40 membrane and modified membranes based on it. Light gray indicates the permeability of the membranes equilibrated with 0.5 M NaCl, dark gray indicates the permeability of the membranes equilibrated with 0.25 M CaCl_2_.

The diffusion permeability of the studied membranes in a 0.5 M NaCl solution and in a 0.25 M CaCl_2_ solution is shown in the Figure 9. In the NaCl solution, unlike the electrical conductivity, it slightly increased with application of the first layer and then slightly decreased with the application of the second layer. For the CaCl_2_ solution, the trend of changes was the same, but their magnitude was much higher.

Several conclusions may be drawn from the obtained results:

1. The electrical conductivity was higher in the NaCl solution than in the equivalent CaCl_2_ solution because the doubly charged counterion bound simultaneously with two singly charged fixed groups [70]. For this reason, its transport within the electric double layers of the membrane decreased.
2. Diffusion permeability, on the contrary, was higher in CaCl_2_ solution than in equivalent NaCl solution. Such observations were made earlier, for example, the authors of [66] deduced the equations to simulate the dependences of diffusion permeability of bilayer membranes on concentration, used them to fit the experimental curves registered for MK-40 membrane, showed that the diffusion permeability of a 2:1 electrolyte depended in a complex way on various physical and chemical parameters, and the diffusion permeability in a 0.5 meg/L NaCl was found to be lower than in 0.5 meg/L CaCl_2_.
  We suggest that one of these factors is more efficient in the screening of functional groups of the membrane by the doubly charged calcium ion than by the singly charged sodium ion. As a result, the thickness of the electric double layer decreased and the pore volume occupied by the electrically neutral solution increased, allowing higher non-selective transport and, hence, diffusion permeability. More efficient screening was confirmed by our measurements of the streaming potential and calculation of the surface charge of MK-40 membrane in solutions containing these ions. However, it should be noted that in [66] a broad range of 1:1 and 2:1 chloride salts were studied and at 0.5 meg/L diffusion permeability increased in row LiCl ≈ NH_4_Cl ≈ NaCl < MgCl_2_ < CaCl_2_ ≈ CsCl < BaCl_2_ ≈ KCl; thus, there also are more important properties than the counterion charge.

Before we discuss changes in electrical conductivity and diffusion permeability occurring after the application of polyelectrolytes let us first consider the porous structure of the membrane substrate and of the applied layers. Several types of pores are present in the structure of MK-40, even when it is a pristine sheet supplied by the manufacturer [37]. There are micropores with a diameter of several nanometers, in which the electric double layers of fixed groups overlap and therefore the electrical current is transported only by counterions, mesopores in which the electric double layers can either overlap or not overlap depending on the concentration of solution, and macropores, in the center of which is an electrically neutral solution, through which ions can be transported non-selectively [71]. Macropores are located between the granules of the ion exchanger and polyethylene [72]. Applied pores may contain micropores and mesopores, but they do not contain macropores.

3. The application of the first modifying layer did not change the electrical conductivity but increased (albeit in a different degree) the diffusion permeability of the membrane. It might be concluded that the properties of the applied layer and of membrane substrate were relatively close, with exception of the diffusion permeability of Ca^2+^ which, as was cited above, strongly depends upon the interaction between the ion and the polymer matrix; for example, the work cited above lists the loss of hydration shell as factor affecting the diffusion permeability. When discussing the closeness of properties, however, it should be noted that the thickness of this layer is more than 100 times smaller than the thickness of substrate membrane, and thus this layer is rather limited in ability to affect the overall properties.
  It should also be noted that we initially supposed that the modification procedure that included drying and exposure to isopropyl alcohol would increase the diameters of pores within the membrane, which in turn would raise both the electrical conductivities and diffusion permeabilities, but electrical conductivities being constant and one of the diffusion permeabilities changing only slightly showed that this effect, even if present, was compensated by the application of polyelectrolyte layer.
4. The decrease in diffusion permeability after applying the second layer, PEI, occurred due to a relatively thick layer of polyelectrolyte that did not contain macropores appearing on the surface of the modified membrane. Assuming that the amount of electroneutral solution in this layer was lower than in MK-40 membrane substrate, then the layer would act as a barrier for non-selective transport of electrolyte, decreasing the diffusion permeability. The fact that the MF-4SC layer did not block the non-selective transport whereas PEI layer did can be explained, in our assumption, by the greater thickness of the latter (judging by Figure 6, the highest estimates of the thickness of the MF-4SC layer were nearly equal to the lowest estimates of thickness of the PEI layer). The hypothesis regarding the PEI layer acting like a barrier for ion transport was supported by the lower electrical conductivity of the MK-40+2 membrane in NaCl solution.

Of particular note was the sharp decrease in the diffusion permeability of CaCl_2_ salt, which occurred with the heterogeneous membrane after the application of the PEI layer, seemingly especially important in the aspect of the creation of monovalent selective membranes.

### 3.4. Current–Voltage Curves

**Figure 10:**
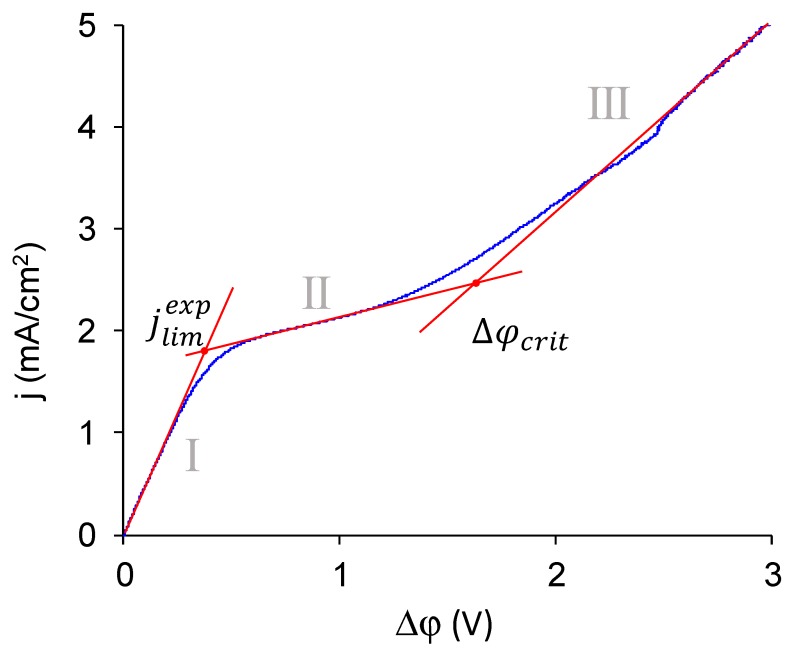
Typical regions in the CVC of a monopolar membrane shown in the CVC of MK-40 membrane in a 0.02 M NaCl solution. Roman numerals indicate the region in the CVC: I is the so-called ohmic region, II is the region of the sloped plateau, III is the region of overlimiting currents. The determination of two points used to compare the membranes, the experimental limiting current density, and the critical potential drop at which the transition to the overlimiting state occurs, is shown.

First, let us discuss the typical CVC of monopolar membrane. The CVC of commercial MK-40 membrane is an example of such (Figure 10). In such CVC, there are three current regions [73,74]. The first, the so-called ohmic region of underlimiting currents, is associated with the formation of concentration profiles inside the diffusion layers in solution near the membrane surface. The second, the region of the sloped plateau, corresponds to a sharp increase in voltage required for the transition from quasi-equilibrium (electrodiffusion, electroconvection occurring by the mechanism of electroosmosis of the first kind [51,75]) to nonequilibrium (generation of H^+^ and OH^−^ ions [76,77], electroconvection occurring by the mechanism of electroosmosis of the second kind [78,79,80]) coupled effects of concentration polarization. The intersection of the tangents built to the initial fragment of the ohmic section and to the plateau region gives the first important point, and the current density of this point is denoted as the experimental limiting current density, *j*_lim_^exp^. The third region, the region of overlimiting currents, appears under the action of coupled effects of concentration polarization. The current density increases here due to the generation of H^+^ and OH^−^ ions, detected when measuring the pH difference between the outlet and the inlet of the desalination chamber, and due to the occurrence of nonequilibrium electroconvection, which might be detected by interferometry [81] and, when it is highly intensive, can be found in potential oscillations found in CVC [82]. The intersection of the tangents drawn to the plateau region and to the overlimiting region gives the second characteristic point of the CVC. The value of the potential drop at this point is designated as the potential drop, Δφ*_crit_*, at which the transition to the overlimiting state occurs.

**Figure 11:**
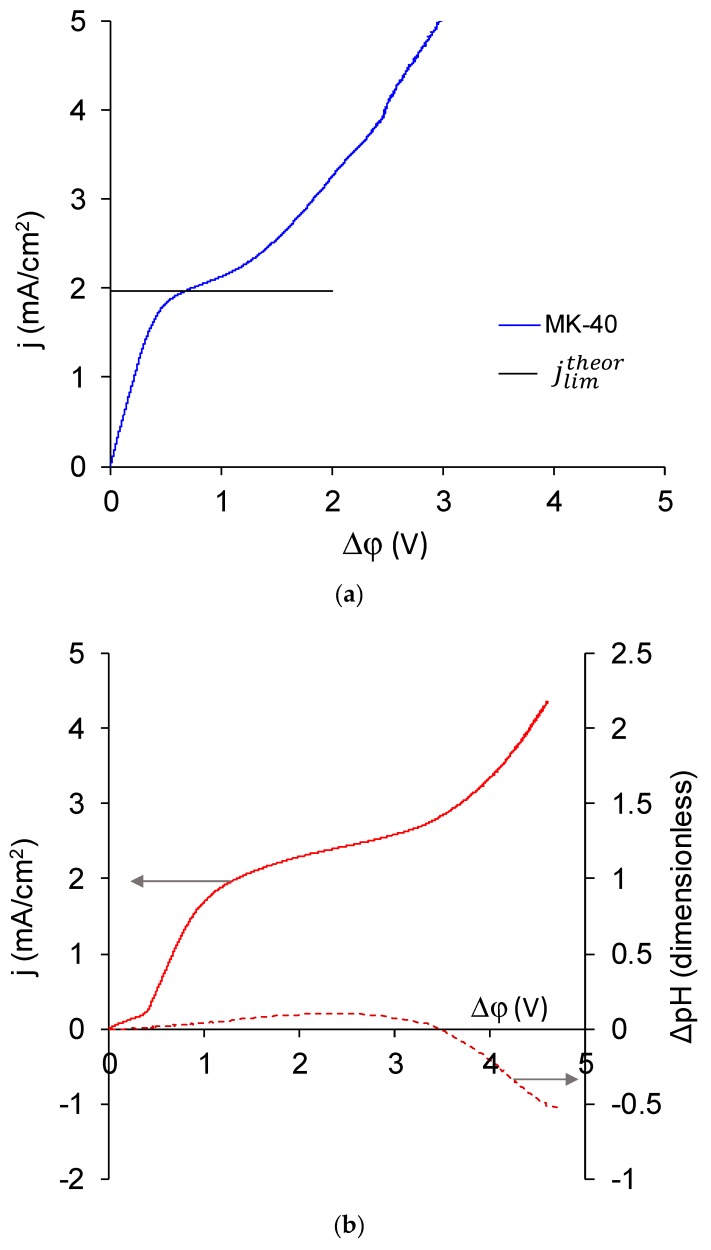

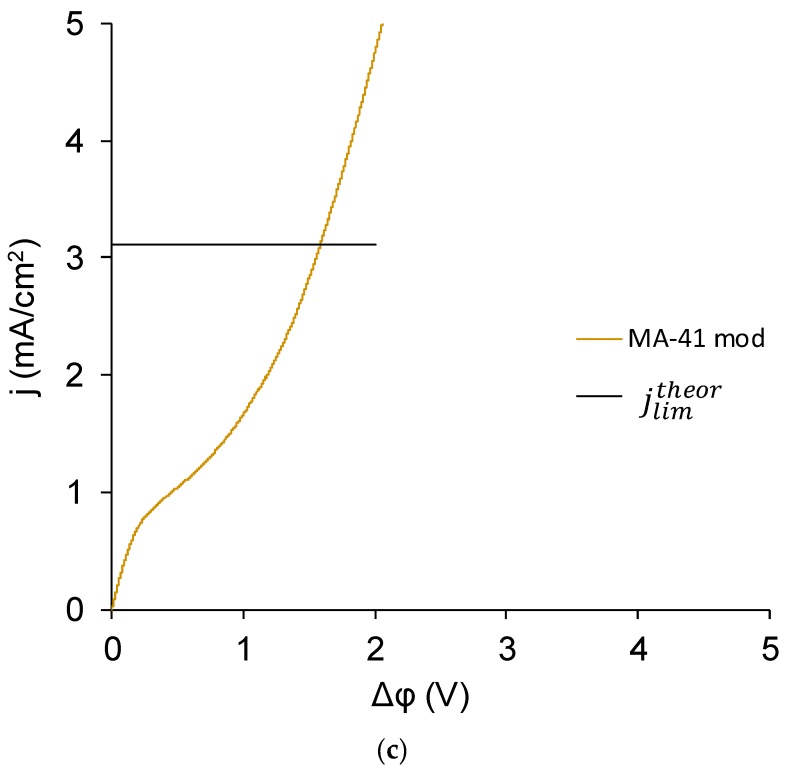
CVC of MK-40 (**a**); MK-40+2 with dependence of pH difference on potential drop over the membrane given in dashed line (**b**); and an asymmetric bipolar membrane—a MA-41 anion exchange membrane modified with a Nafion cation exchange layer, which is given for comparison (**c**). All curves were registered in a 0.02 M NaCl solution. For our system, the theoretical limiting current calculated by the Lévêque equation for cation exchange (MK-40) membrane was 1.96 mA/cm^2^ and for anion exchange (MA-41) membrane was 3.00 mA/cm^2^.

In the case of the MK-40+2 membrane, two extreme cases of the CVC, as well as any intermediate case, were possible. The first extreme case corresponds to a monopolar (Figure 11a) and the second to a (asymmetric) bipolar membrane (Figure 11c). If the presence of the modifying layer did not affect the transport of salt counterions (in this case, Na^+^ ions) and did not lead to the appearance of intensive generation of H^+^ and OH^−^ ions in underlimiting currents, the CVC of the MK-40+2 membrane would have a typical form for a monopolar membrane and would approximately match a CVC of MK-40 membrane. If the thickness of the adsorbed layer were large, then the transport of salt ions would be blocked, the membrane would become bipolar, and its CVC would take the form shown in Figure 11c [43]. In the latter case, the solution in the desalination chamber would get alkalized, which corresponds to more intensive generation of H^+^ and OH^−^ ions on the cation exchange membrane; in the opposite case of more intensive generation of H^+^ and OH^−^ ions on the anion exchange membrane, the solution would begin to acidify, and this usually happens when the voltage in membrane system increases because the amino groups that are present in the majority of commercial anion exchange membranes [83] are more catalytically active in the water dissociation reaction [77] compared with the sulfonic groups of the majority of commercial cation exchange membranes.

In reality, the CVC of the MK-40+2 membrane (Figure 11b) was found to be more similar to the CVC of a monopolar membrane, but there were two differences.

The first is the presence of an initial inclined section, which we associate with the desalination of the modifying layer. Its mechanism is as follows: as the transport of Na^+^ ions through the cation exchange membrane and Cl^–^ ions through the PEI layer to the bipolar boundary was blocked by the applied external electric field, and the transport of Na^+^ ions through the PEI layer and Cl^–^ ions through the cation exchange membrane was hindered by their electrostatic interactions with fixed groups, then the passage of current through this system started desalination at the bipolar boundary, which spread over time to the bulk of polyelectrolytes and to the membrane bulk.

The second is a presence of a noticeable alkalization of the solution, which began in underlimiting current modes and was changed to acidification at higher potential drops. The alkalinization indicated the generation of H^+^ and OH^−^ ions occurring at the bipolar boundary, and therefore showed that the bipolar boundary will play a role in the resulting properties of the modified membrane. The resulting shape of dependence of pH difference on potential drop is explained as follows. For a bilayer membrane with the cation exchange side facing the cathode and the (supposedly) anion exchange side facing the anode, the bipolar boundary is the fastest desalted place in the system, as transport numbers of the respective counterions through charged layers are higher than through the solution. This place is the first to reach the limiting state and to start the generation of H^+^ and OH^−^ ions. Produced H^+^ ions were transferred in the direction of the cathode through cation exchange membranes into the auxiliary chamber, and OH- ions were transferred through the PEI layer in the direction of the anode and into the desalination chamber. Glass electrode mounted in the desalination channel past the desalination chamber detected alkalization. According to the Peers equation, if the transport number of cation in solution is lower than the transport number of anion in solution, as it is for studied 0.02 M NaCl and 0.01 M CaCl_2_ solutions, then with increasing current density the limiting state is reached at membrane/solution boundary of cation exchange membrane earlier than at the same boundary of anion exchange membrane. The generation of H^+^ and OH^–^ ions at modified cation exchange/solution boundary also leads to alkalification. Finally, the limiting state was reached at anion exchange membrane that forms another wall of desalination chamber, and there OH^–^ ions were transferred in the direction of anode through the anion exchange membrane and H^+^ ions remained in desalination chamber. Depending on relative intensities of generation of H^+^ and OH^−^ ions at the cation and anion exchange membranes, which in turn depends, for example, on the nature of functional groups of these membranes [81], the pH may either continue to grow or start falling. Because we observed acidification, we can conclude that at high current densities and potential drop, the generation of H^+^ and OH^−^ ions was more intensive at the anion exchange membrane.

On the basis of the CVC, it can be concluded that the MK-40+2 membrane was largely monopolar; however, at all currents it combined two functions: the dominant separation of cations and the less pronounced generation of H^+^ and OH^–^ ions.

**Figure 12:**
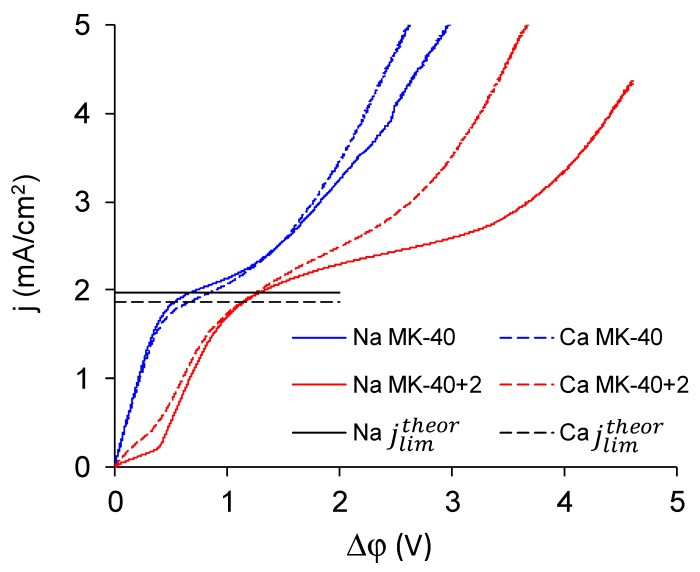
CVC of the MK-40 (blue lines) and the MK-40+2 (red lines) membranes registered in 0.02 M NaCl solution (solid lines) and 0.01 M CaCl_2_ solution (dashed lines). Black lines denote the theoretical limiting current densities calculated by the Lévêque equation: the solid black line shows the limiting current density of Na^+^ (1.96 mA/cm^2^) and the dashed black line shows the limiting current density of Ca^2+^ (1.83 mA/cm^2^).

Comparison of CVC registered in NaCl and in CaCl_2_ solution of the same equivalent concentration (0.02 meg/L) showed the response of the system on the change of electrolyte (Figure 12). Parameters of characteristic points are summed in Table 2.

**Figure 13:**
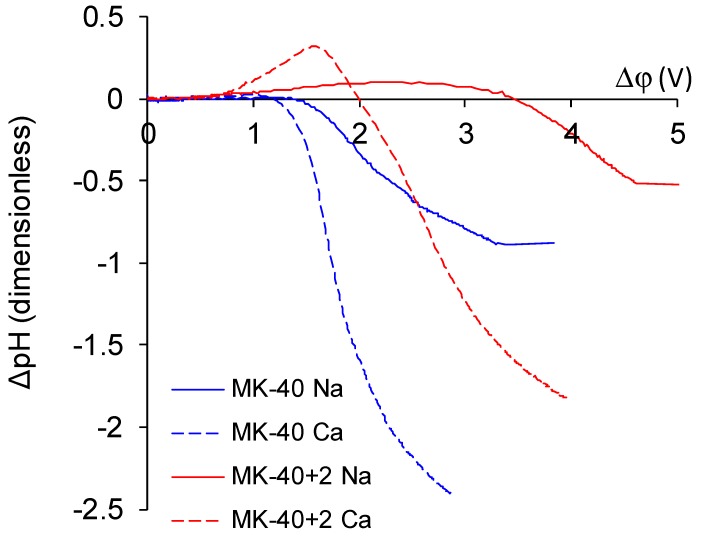
The dependence of pH changes in the desalination channel on the potential drop at the membrane.

The following patterns can be identified on the basis of these CVCs:

- The experimental limiting current densities of membranes in solutions of the same equivalent concentration but with different charge number of counterions were close to each other and to the respective theoretical limiting current densities, and the experimental limiting current densities became even closer to each other after being normalized to the theoretical limiting current densities. Because the Lévêque equation used for the calculation of the theoretical limiting current density does not consider the membrane structure or the specific interactions between a salt and a membrane, the good agreement of the experiment with this calculation meant that the used membranes conducted the Na^+^ and Ca^2+^ equally well, which meant that modification does not particularly hinder the transport of a doubly charged ion. Appearance of such hindering was expected because the ultimate goal of the modifications, similar to that done in this work, was improvement of the monovalent selectivity through the repulsion of multicharged ions. It can be concluded that the application of a single modifying layer does not yet make detectable changes in the selectivity of the membrane.
- The overlimiting mode of the modified membrane started at significantly higher potential drops than that of the nonmodified membrane. This seems somewhat unexpected, given the fact that the generation of H^+^ and OH^−^ ions, which in itself is one of the major mechanisms of overlimiting transport, arose for the modified membrane even in underlimiting current modes (Figure 11c and Figure 13). In addition, a higher roughness of its surface should enhance the development of another major mechanism of an overlimiting increase in mass transport—electroconvection [59,84,85]. It can be assumed that the reason for the observed delay was the suppression of electroconvection by the low-intensity generation of H^+^ and OH^−^ ions, as it is known that the coions produced in this reaction partially destroy [86] the extended space charge region necessary for the development of electroconvection by the mechanism of electroosmosis of the second kind [79,87]. Another proof for this explanation is that for modified membranes, the potential drop of transition to overlimiting state was close to the potential drop of the onset of more intensive generation of H^+^ and OH^−^ ions at the anion exchange membrane (Figure 13), which started supplying H^+^ ions in the desalination channel. These H^+^ ions were additional charge carriers themselves and they neutralized OH^–^ ions, which were destroying the extended space charge region of the cation exchange membrane.
- The slope of the overlimiting region of the CVC registered in the CaCl_2_ solution was higher, and the critical potential drop of the transition to the overlimiting state in this solution was at least comparable to such a potential drop in the NaCl solution (in the case of the MK-40 membrane) or much lower than it (in the case of the MK-40+2 membrane). We attribute this to a more intensive development of electroconvection, as Ca^2+^ ion is more hydrated than Na^+^ ion and, as a result, it involves a larger volume of solution in motion at equal equivalent concentration [88,89].

## 4. Conclusions

The surface of the MK-40 heterogeneous cation exchange membrane was coated with the homogenizing cation exchange layer and the layer carrying amino groups, and then the change in membrane properties was studied when treating the NaCl and CaCl_2_ solutions at neutral pH (6–7). It was shown that although part of amino groups may be protonated, the limiting current of the membrane was preserved, non-selective ion transfer was reduced, and the primary function of the membrane was still the transport of salt counterions and not the generation of H^+^ and OH^−^ ions. A number of undesirable changes was also observed, such as a decrease in the electrical conductivity and the appearance of the generation of H^+^ and OH^−^ ions even in underlimiting current modes, however, they apparently did not significantly affect the properties of the modified sample. This allows us to recommend such a substrate and an approach to modification for the further creation of inexpensive membranes with monovalent selectivity.

An important discovery is that even in the case when the top layer of the modifier was made of anion exchange material, the surface charge of the membrane remained negative. Apparently this was due to the fact that the only about a quarter of nitrogen in polyethyleneimine was protonated in the neutral pH range. The switch from such weakly basic material to materials containing quaternary ammonium bases charged in a much wider pH range might be beneficial for barrier properties and monovalent selectivity of the created materials.

## Figures and Tables

**Table 1:** Experimental dependences of the streaming potentials on overpressure, calculated zeta potentials, and membrane surface charges. Subscript N denotes the zeta potentials and surface charges calculated when taking into account the membrane roughness.

System	ΔE/Δp, mV/bar	ζ, mV	σ, µC/cm^2^	ζ_N_, mV	σ_N_, µC/cm^2^
MK-40 in 0.002 M NaCl	−3.5	−171	−24.99	−165.6	−22.45
MK-40+2 in 0.002 M NaCl	−0.7	−104	−6.50	−98	−5.74
MK-40 in 0.02 M NaCl	−0.25	−49	−1.90	−47.5	−1.8
MK-40+2 in 0.02 M NaCl	−0.08	−39.6	−1.46	−37.3	−1.36
MK-40 in 0.001 M CaCl_2_	−1.5	−44	−1.66	−42.6	−1.59
MK-40+2 in 0.001 M CaCl_2_	−0.2	−17.5	−0.59	−16.5	−0.56
MK-40 in 0.01 M CaCl_2_	−0.14	−14.3	−0.48	−13.9	−0.47
MK-40+2 in 0.01 M CaCl_2_	−0.05	−15.3	−0.52	−14.4	−0.49

**Table 2:** The main parameters of the characteristic points of the CVC of the commercial MK-40 membrane and the modified MK-40+2 membrane created on its basis. Theoretical limiting current densities are calculated by the Lévêque equation.

Property	MK-40	MK-40+2
Experimental limiting current density $i_{lim}^{exp}$ in 0.02 M NaCl, mA/cm^2^	1.81	1.99
Experimental limiting current density $i_{lim}^{exp}$ in 0.01 M CaCl_2_, mA/cm^2^	1.69	1.78
Theoretical limiting current density $i_{lim}^{theor}$ in 0.02 M NaCl, mA/cm^2^	1.96	1.96
Theoretical limiting current density $i_{lim}^{theor}$ in 0.01 M CaCl_2_, mA/cm^2^	1.83	1.83
$i_{lim}^{exp}/i_{lim}^{theor}$ in 0.02 M NaCl	0.92	1.01
$i_{lim}^{exp}/i_{lim}^{theor}$ in 0.01 M CaCl_2_	0.91	0.96
Critical potential drop of transition to overlimiting state $\Delta \varphi_{crit}$ in 0.02 M NaCl, V	1.63	3.75
Critical potential drop of transition to overlimiting state $\Delta \varphi_{crit}$ in 0.01 M CaCl_2_, V	1.60	2.86

## Supplementary Materials

The following are available online at https://www.mdpi.com/2077-0375/10/2/20/s1. AFM reports.
