# Supplementary figures and images for: Characterization of MK-40 Membrane Modified by Layers of Cation Exchange and Anion Exchange Polyelectrolytes
Source: Membranes (Basel). 2020 Jan 27;10(2):20. doi: 10.3390/membranes10020020 (PMC7073548; doi:10.3390/membranes10020020)

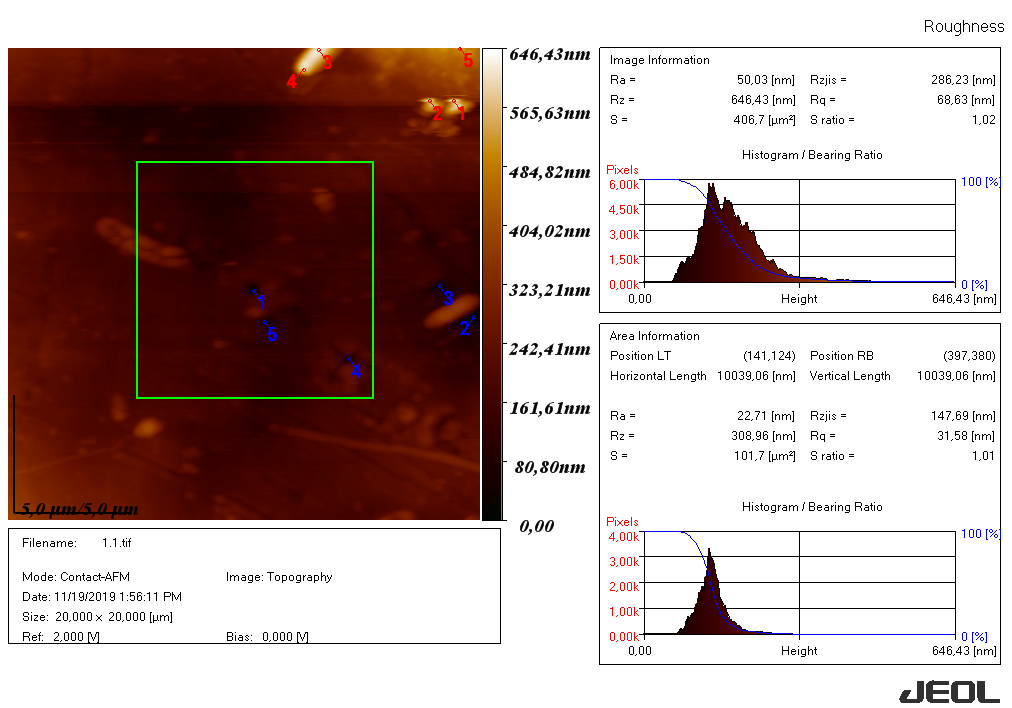

Supplement: Supplementary file 1 [file membranes-10-00020-s001.zip › AFM reports/1.1 (S).png]

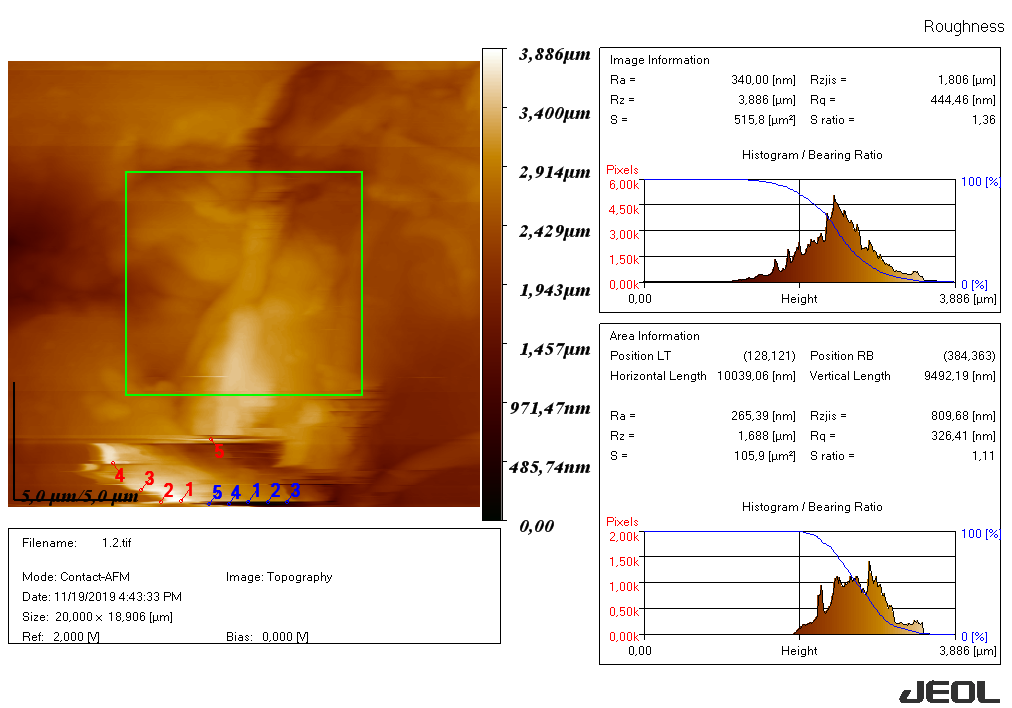

Supplement: Supplementary file 1 [file membranes-10-00020-s001.zip › AFM reports/1.2 (S).png]

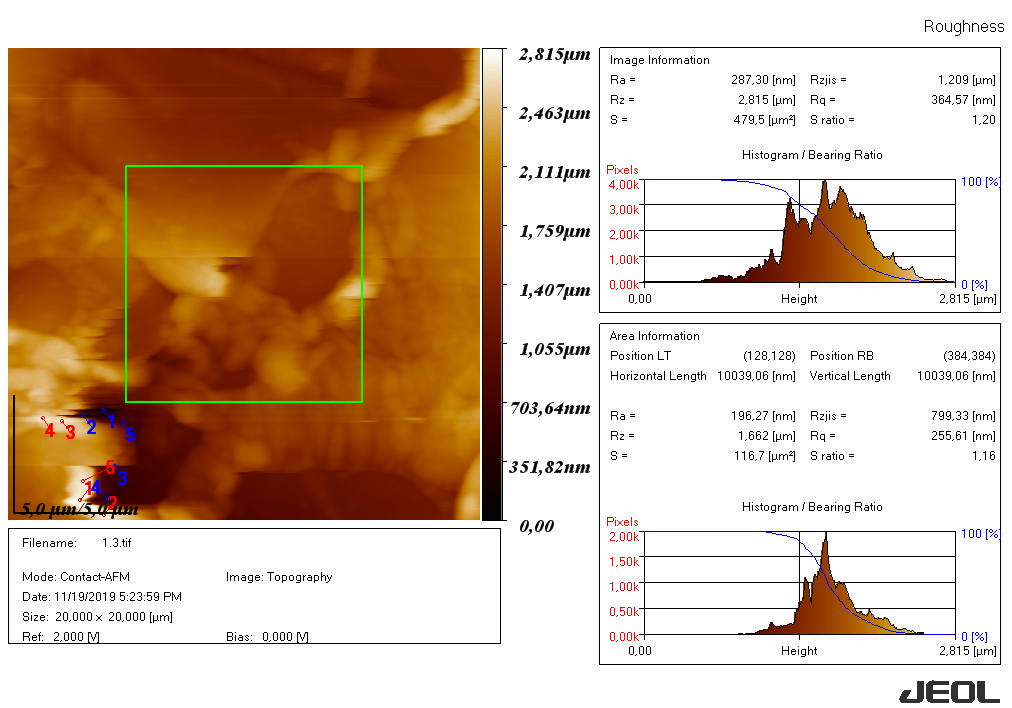

Supplement: Supplementary file 1 [file membranes-10-00020-s001.zip › AFM reports/1.3 (S).png]

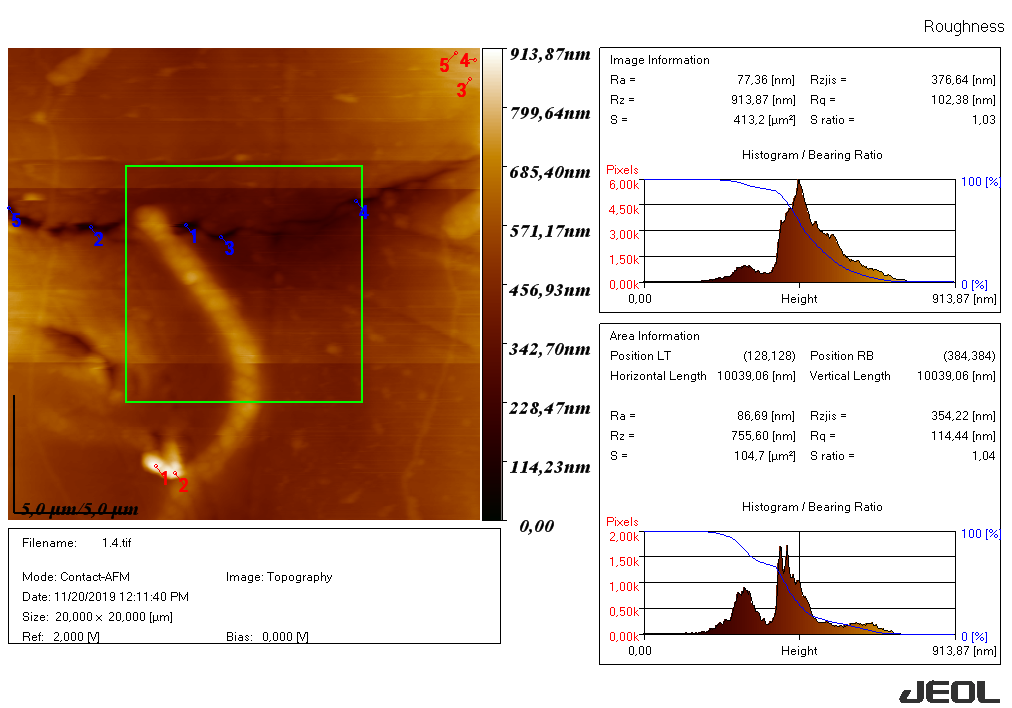

Supplement: Supplementary file 1 [file membranes-10-00020-s001.zip › AFM reports/1.4 (S).png]

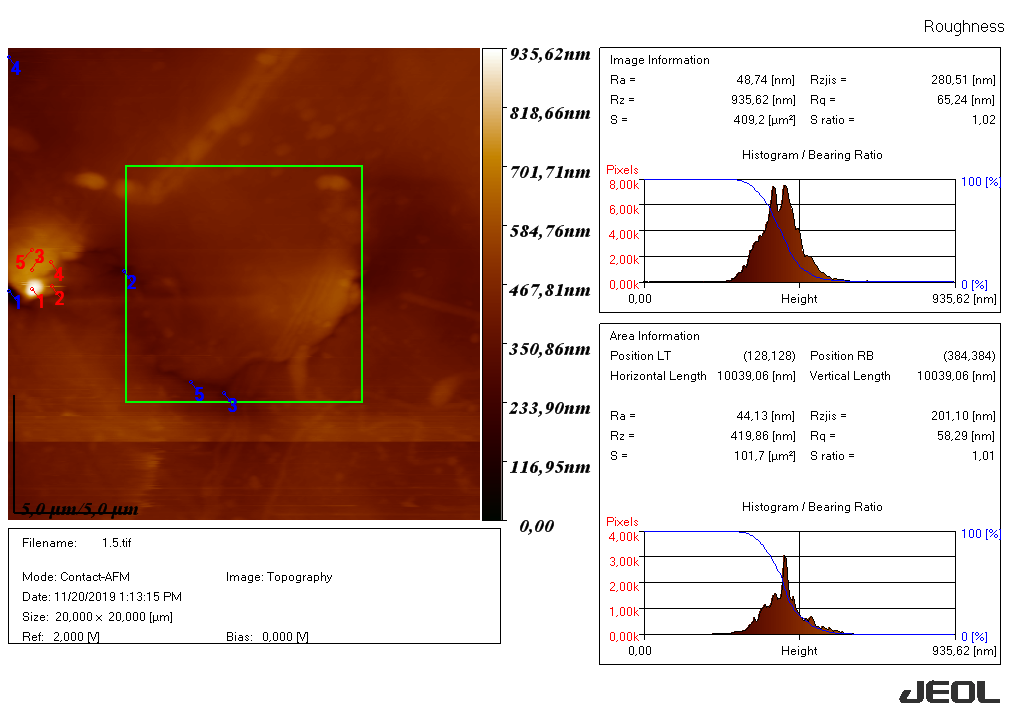

Supplement: Supplementary file 1 [file membranes-10-00020-s001.zip › AFM reports/1.5 (S).png]

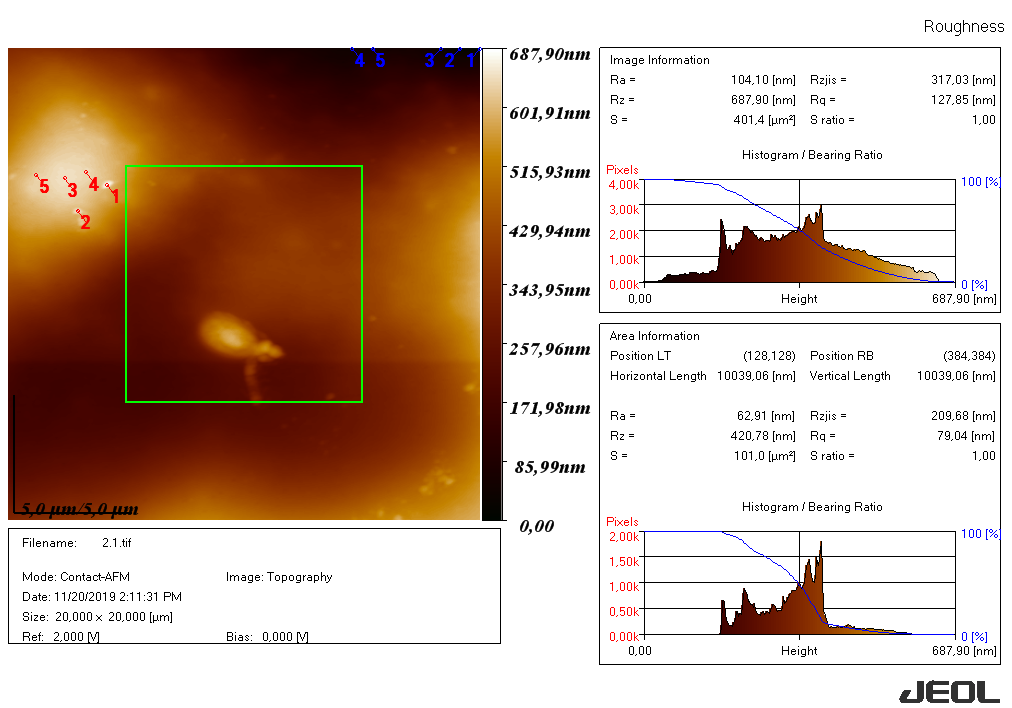

Supplement: Supplementary file 1 [file membranes-10-00020-s001.zip › AFM reports/2.1 (S).png]

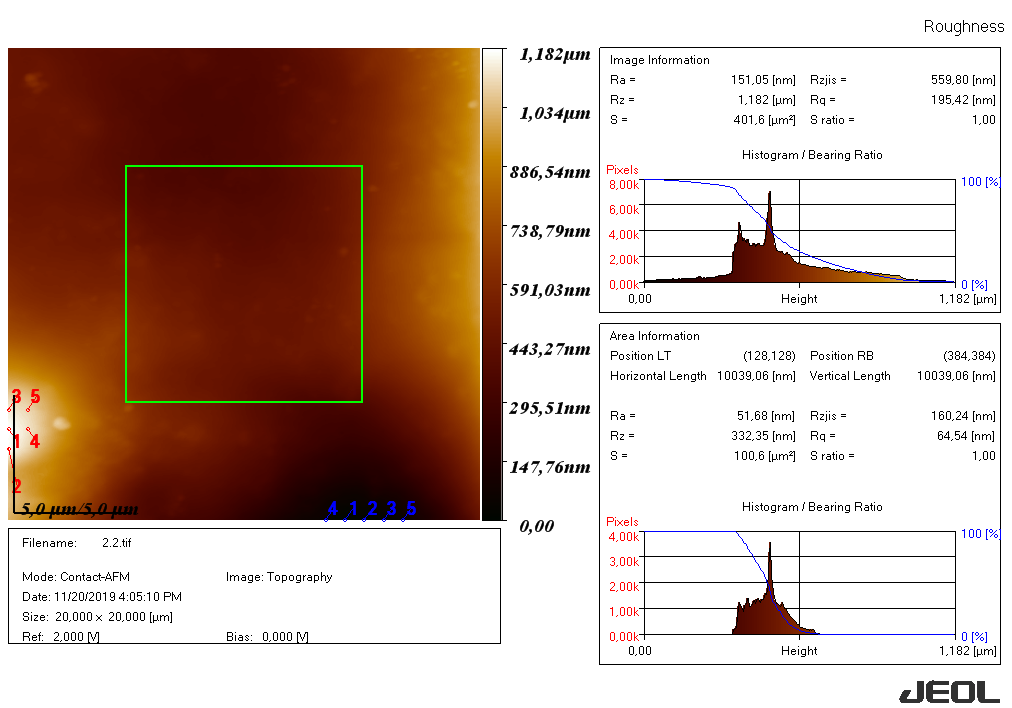

Supplement: Supplementary file 1 [file membranes-10-00020-s001.zip › AFM reports/2.2 (S).png]

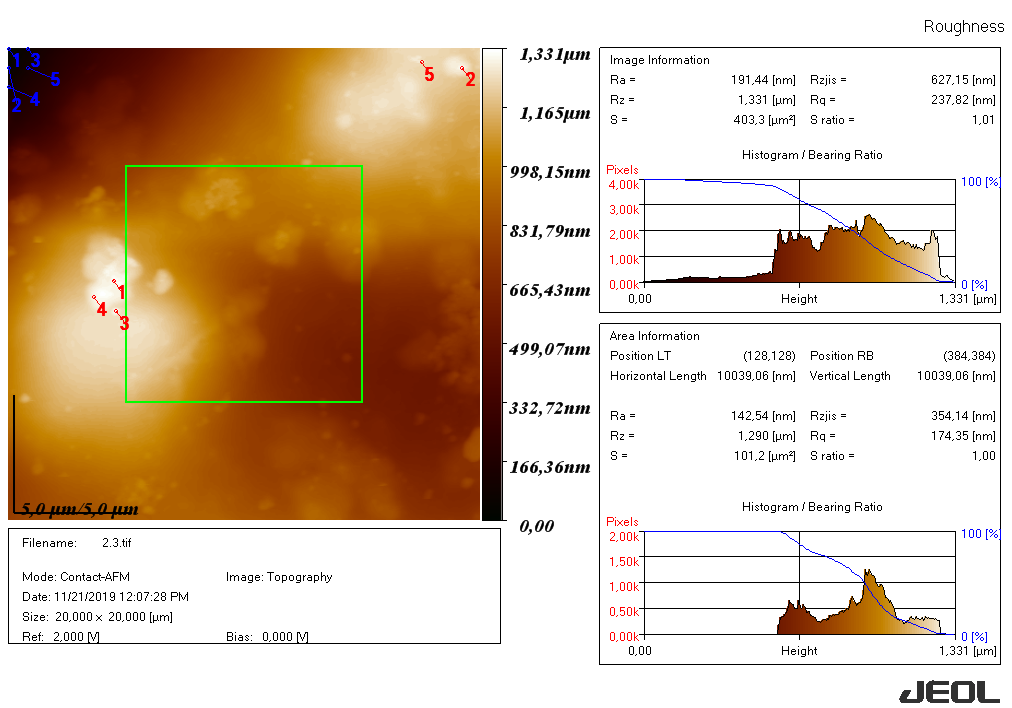

Supplement: Supplementary file 1 [file membranes-10-00020-s001.zip › AFM reports/2.3 (S).png]

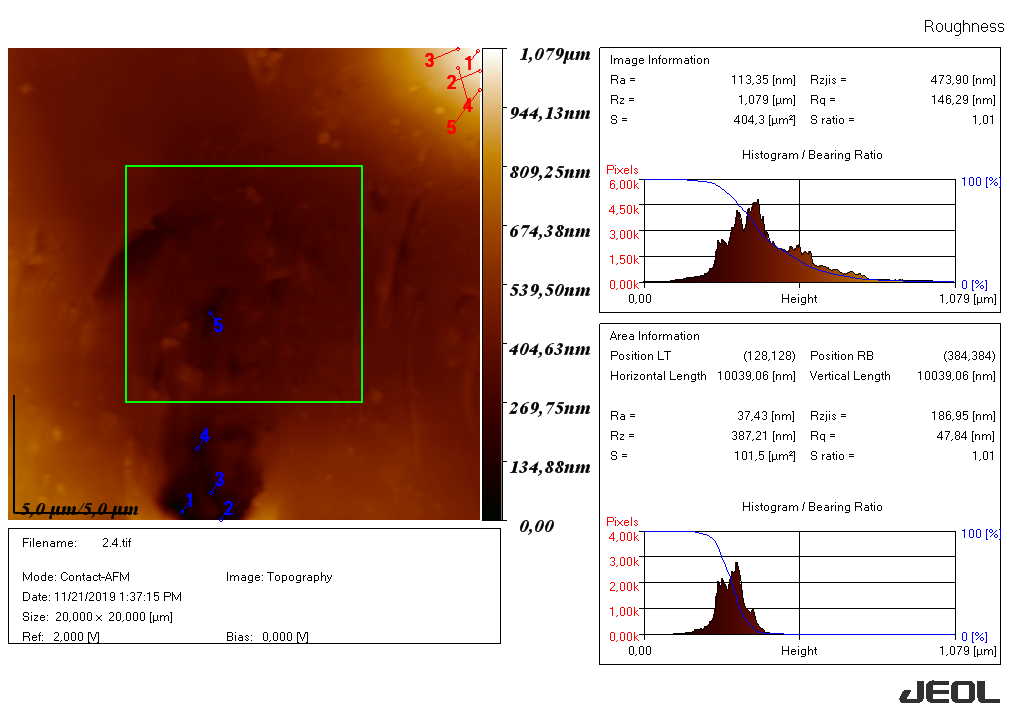

Supplement: Supplementary file 1 [file membranes-10-00020-s001.zip › AFM reports/2.4 (S).png]

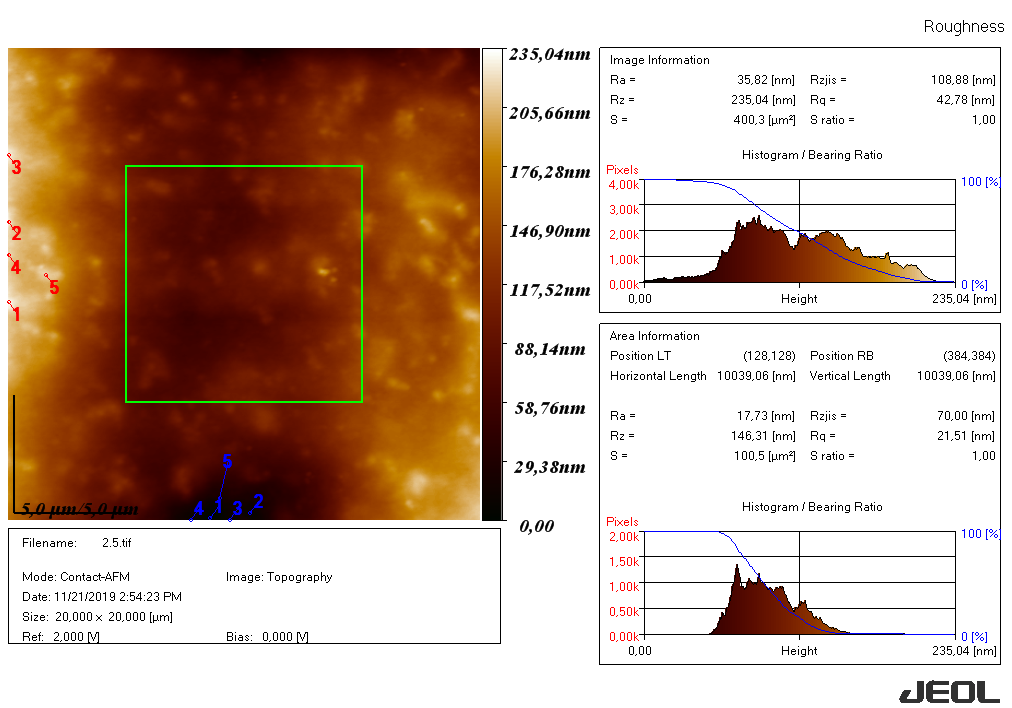

Supplement: Supplementary file 1 [file membranes-10-00020-s001.zip › AFM reports/2.5 (S).png]

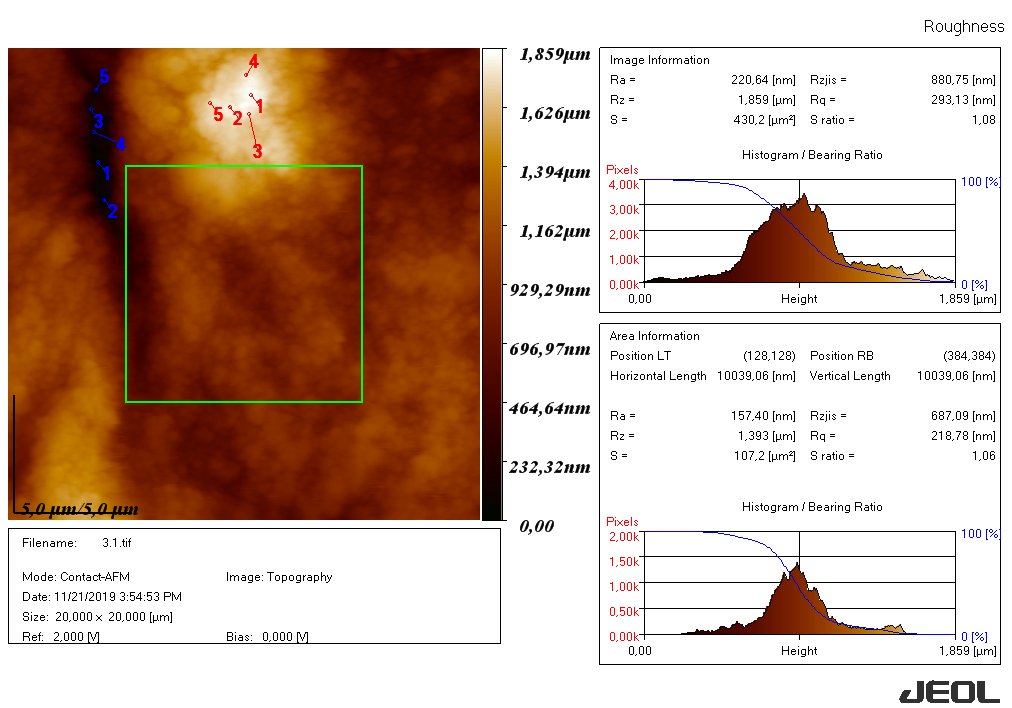

Supplement: Supplementary file 1 [file membranes-10-00020-s001.zip › AFM reports/3.1 (S).png]

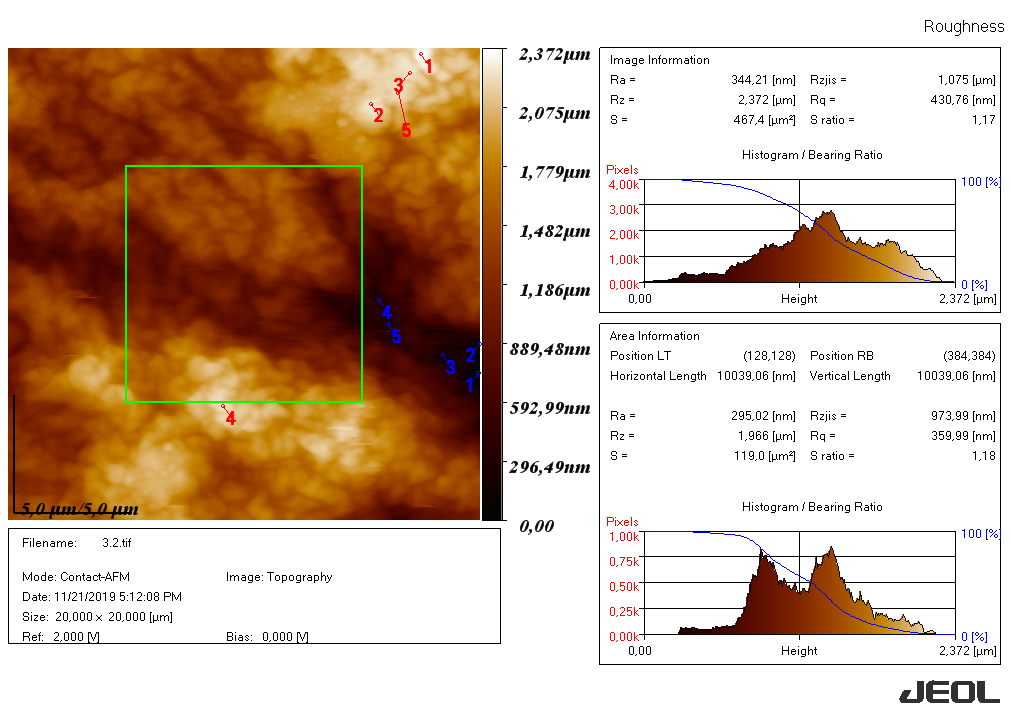

Supplement: Supplementary file 1 [file membranes-10-00020-s001.zip › AFM reports/3.2 (S).png]

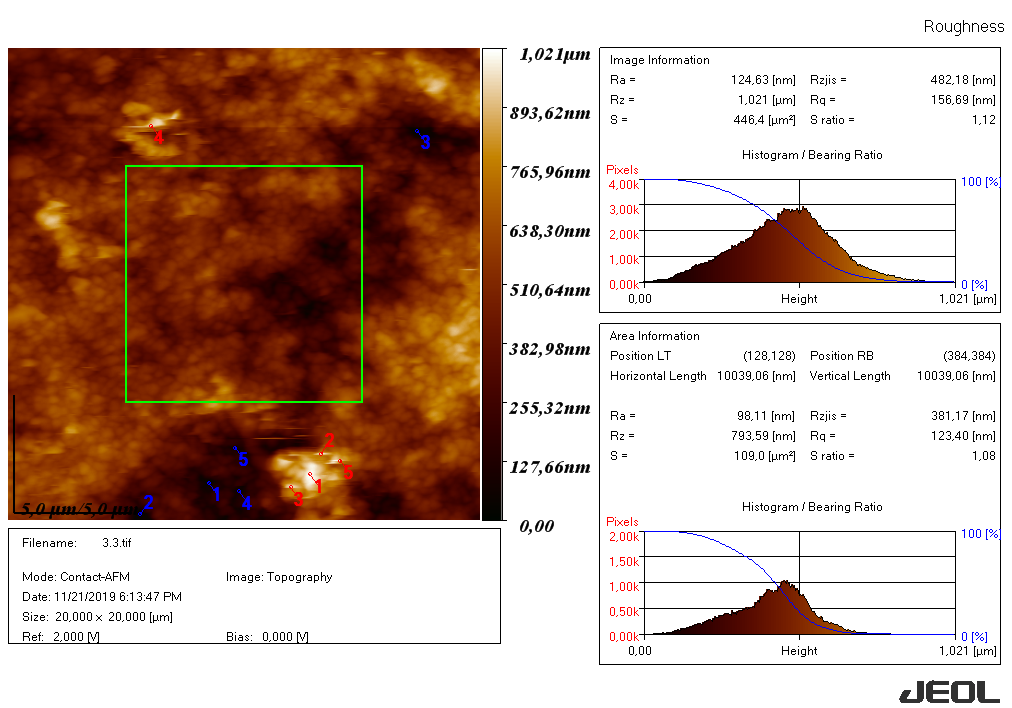

Supplement: Supplementary file 1 [file membranes-10-00020-s001.zip › AFM reports/3.3 (S).png]

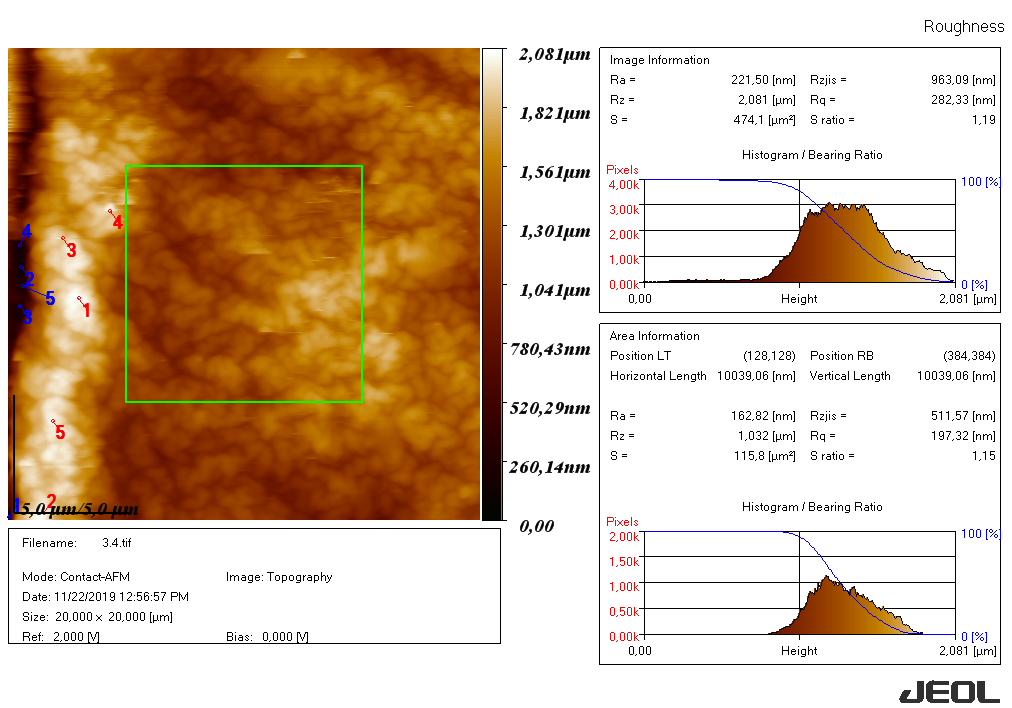

Supplement: Supplementary file 1 [file membranes-10-00020-s001.zip › AFM reports/3.4 (S).png]

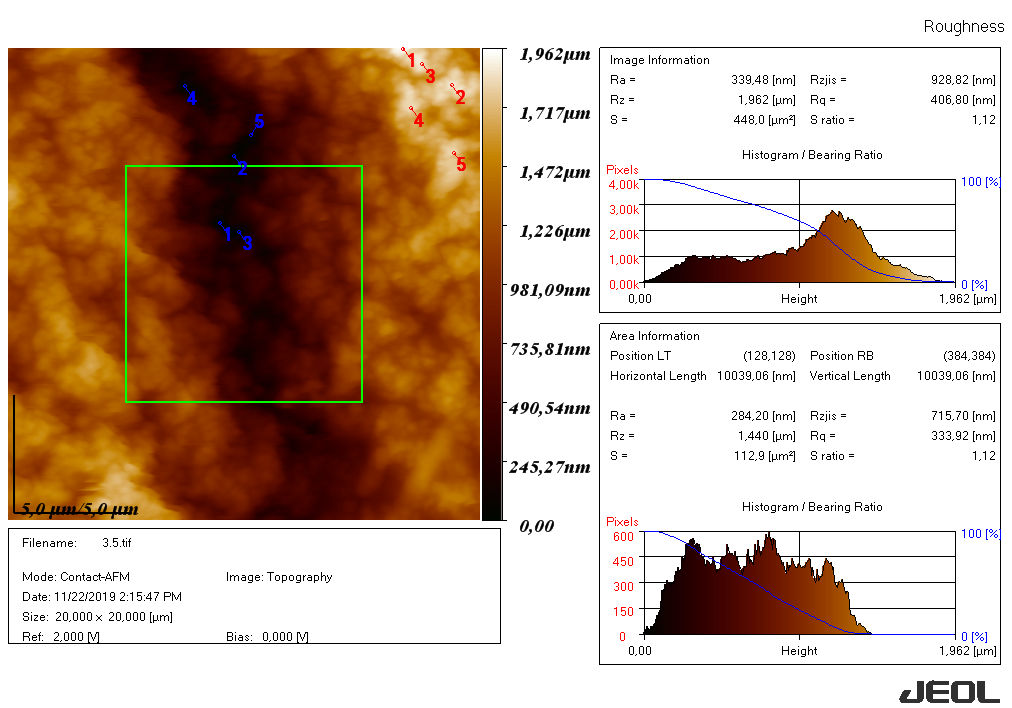

Supplement: Supplementary file 1 [file membranes-10-00020-s001.zip › AFM reports/3.5 (S).png]
